# Theoretical Investigation of Adsorption of Eugenol and Methyleugenol on the Coronene Surface: A Simplified Finite Molecular Model of Graphene

**DOI:** 10.1021/acsomega.6c07744

**Published:** 2026-10-09

**Authors:** Yuri dos Santos Lima Pereira, Jonas Marinho Duarte, Alcy Favacho Ribeiro, Josiney Farias de Araújo, Alessandra Nacimento Braga, Marcos Vinícius da Silva Paula, Carlos Alberto Brito da Silva Jr

**Affiliations:** † Faculdade de Química, 37871 UFPA, Ananindeua 67113-901, Brazil; ‡ Faculdade de Engenharia de Minas, UFOPA, Juruti 68170-000, Brazil; § Pós-Graduação Em Botânica Tropical, UFRA, Belém 66077-530, Brazil; ∥ Faculdade de Física, UFPA, Ananindeua 67113-901, Brazil; ⊥ Faculdade de Engenharia de Materiais, UFPA, Ananindeua 67113-901, Brazil

## Abstract

Graphene-based materials have attracted considerable interest for molecular adsorption, chemical sensing, and protective applications because of their extended π-conjugated electronic structure and ability to interact with aromatic compounds. Herein, a comparative first-principles investigation was performed on the adsorption of eugenol (C_10_H_12_O_2_) and methyleugenol (C_11_H_14_O_2_) on coronene (C_24_H_12_), employed as a simplified finite molecular model of a local graphene-like surface. Density functional theory calculations were performed using the ωB97X-D3/6–31G­(d) approach, complemented by alternative functionals and the def2-TZVPD basis set to assess basis-set effects and basis set superposition error (BSSE). Both molecules preferentially adopted parallel configurations relative to the coronene surface, with adsorption distances characteristic of physisorption. The uncorrected adsorption energies were −0.645 and −0.672 eV for eugenol and methyleugenol, respectively, whereas BSSE-corrected benchmark calculations with def2-TZVPD yielded values between −0.536 and −0.593 eV. Energy decomposition analysis (EDA) identified dispersion as the dominant stabilizing contribution, followed by electrostatic and orbital interactions. Charge-transfer, molecular electrostatic potential, frontier molecular orbital, and density-of-states analyses revealed weak electronic coupling and limited charge redistribution upon adsorption. Vibrational calculations showed no imaginary frequencies and only minor changes in IR and Raman features, supporting the stability and predominantly noncovalent character of the complexes. Overall, methylation of the phenolic hydroxyl group produced a small increase in adsorption strength within the coronene model without changing the same physisorption mechanism. These results provide molecular-level insights into phenylpropanoid interactions with graphene-like carbon surfaces.

## Graphical abstract

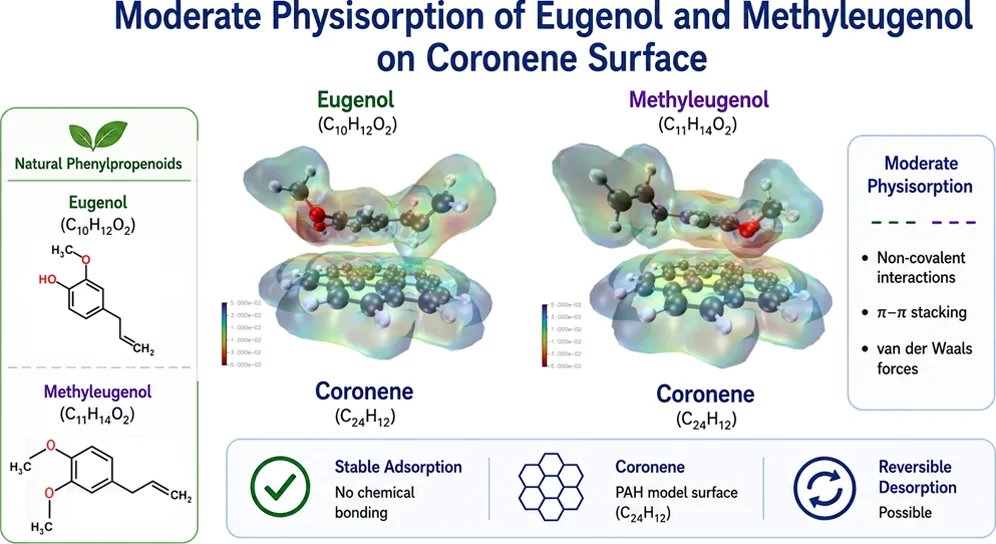

## 1 Introduction

Graphene is a two-dimensional (2D) sp^2^-hybridized carbon allotrope characterized by a highly conjugated π-electron system, exceptional mechanical strength, high electrical conductivity, and distinctive surface properties.^1–3^ Since its isolation, graphene and related carbon-based materials have attracted considerable interest for applications in electronics, energy storage, catalysis, sensing, separation, and surface protection.^2–5^ In particular, the delocalized π-electron system of graphene can interact favorably with aromatic and π-electron-rich molecules through a combination of dispersion, π–π, electrostatic, and, depending on the chemical functionality, hydrogen-bonding interactions. These interactions are relevant to adsorption processes and to the development of graphene-based chemical sensors, in which molecular adsorption can alter the electronic structure and charge-transport characteristics of the sensing material.^4,5^ Understanding the interaction between graphene-like surfaces and functional organic molecules at the molecular level is therefore important for the rational design of adsorption, sensing, and surface-functionalization strategies.

The physicochemical properties of graphene are also relevant to surface protection and corrosion control. Although graphene itself is a nonmetallic carbon material and does not undergo corrosion in the conventional electrochemical sense applicable to metallic substrates, its structural integrity, chemical stability, and low permeability can allow it to act as a barrier against the transport of water, oxygen, and aggressive species.^6^ Experimental studies have demonstrated that graphene coatings can reduce the electrochemical degradation of metallic substrates, while broader investigations have established the potential of graphene-based materials in anticorrosion coatings.^7,8^ The high intrinsic strength of graphene,^9^ together with its low permeability and chemical stability, further supports its application as a protective component in multifunctional coatings.

Nevertheless, the interaction of graphene and graphene-derived materials with their chemical environment is more complex than simple physical impermeability. Graphene oxide, for example, contains oxygenated functional groups that substantially modify its chemical reactivity and susceptibility to environmental degradation.^10,11^ Moreover, the protective performance of graphene coatings can depend strongly on defects, interfaces, substrate interactions, and localized pathways for the penetration of corrosive species.^8,12,13^ These characteristics have motivated the development of hybrid graphene-based materials in which the carbon surface is combined with molecular or polymeric components to provide additional chemical functionality, hydrophobicity, and corrosion protection.^11,13,14^ Consequently, understanding the molecular–scale interaction between aromatic organic compounds and π-conjugated carbon surfaces is relevant to elucidating the fundamental processes governing such hybrid interfaces.

Among naturally occurring aromatic compounds, phenylpropanoids have received considerable attention because of their biological, chemical, and technological properties. Eugenol (C_10_H_12_O_2_), the principal phenylpropanoid constituent of clove (*Syzygium aromaticum*) essential oil, contains an aromatic ring substituted with a hydroxyl group (−OH), a methoxy group (−OCH_3_), and an allyl moiety (CH_2_CH=CH_2_).^15,16^ Eugenol and related phenylpropanoids also occur in several plants and essential oils, including species of the genus *Piper*, which are particularly relevant to the biodiversity of the Amazon region.^17^ Methyl eugenol (C_11_H_14_O_2_), a structurally related derivative of eugenol, differs primarily through methylation of the phenolic hydroxyl group converting a phenol (Ar–OH) into aryl methyl ether (Ar-OCH_3_). Although this modification is chemically simple, it changes the hydrogen bonding capability, polarity, and electronic distribution of the molecule and may consequently influence its interaction with carbonaceous surfaces.

The chemical functionality of eugenol and methyleugenol is particularly relevant to adsorption and corrosion inhibition. Oxygen-containing functional groups and the aromatic π-electron system can contribute to the interaction of these molecules with metallic and carbon-based surfaces. Eugenol derivatives have been experimentally investigated as corrosion inhibitors for steel in acidic media, demonstrating the relevance of molecular structure to surface adsorption and inhibition efficiency.^18^ More broadly, phenylpropanoids are recognized for their antioxidant properties and their ability to participate in diverse chemical and biological processes.^19,20^ Essential oils containing eugenol and related compounds have consequently been investigated as potentially environmentally attractive corrosion inhibitors.^16,21,22^ Recent work on *Piper divaricatum* essential oil, for example, has further highlighted the anticorrosive potential of plant-derived compounds from the Amazon region.^23^

The incorporation of eugenol-derived compounds into graphene-based materials provides an additional motivation for investigating their molecular interactions. Graphene and graphene oxide can act as physical barriers against water, oxygen, and aggressive ions, while organic components can introduce additional chemical functionality and contribute to the formation of protective interfacial films.^6,11,13,14^ Polymerized eugenol derivatives and graphene-based materials have therefore attracted interest as components of multifunctional and potentially more sustainable protective systems.^11,14,21,22^ However, the macroscopic performance of such hybrid materials ultimately depends on molecular–scale interactions between their components. In particular, adsorption geometry, interaction strength, charge redistribution, and electronic coupling between an aromatic molecule and a π-conjugated carbon surface can determine the stability and functionality of the resulting interface.

At the molecular level, adsorption of aromatic compounds on graphene-like surfaces can involve several noncovalent contributions, including π–π interactions between the molecular aromatic ring and the carbon π-system, dispersion interactions, electrostatic interactions, and, depending on the molecular functional groups and adsorption configuration, hydrogen bonding and charge transfer. The relative contribution of these interactions can be strongly affected by molecular functionalization. Consequently, comparing structurally related molecules provides a useful strategy for identifying how specific functional groups influence adsorption energetics and electronic properties. In the case of eugenol and methyleugenol, conversion of the phenolic hydroxyl group into a methoxy group provides a well-defined structural modification for investigating the influence of hydrogen-bonding capability and molecular electronic structure on adsorption at a π-conjugated carbon surface.

First-principles calculations based on density functional theory (DFT) provide an appropriate framework for investigating these effects at the atomic and electronic levels. Such calculations can provide information on adsorption energies (E_Ads_), equilibrium geometries, charge redistribution, electronic states, frontier molecular orbitals, and vibrational properties, thereby allowing the nature of adsorbate–surface interactions to be characterized beyond what can be directly inferred from macroscopic experiments. For extended graphene sheets, however, periodic models containing large numbers of atoms can become computationally demanding, particularly when several adsorption configurations and vibrational properties are considered. Finite molecular models based on polycyclic aromatic hydrocarbons (PAHs) can therefore provide a computationally tractable approach for examining local interactions involving extended π-conjugated carbon frameworks.

Recent first-principles studies have further demonstrated the importance of noncovalent interactions in the adsorption of biologically relevant aromatic molecules on graphene. Tayo et al.^24^ investigated the adsorption of the DNA nucleobases adenine, guanine, thymine, and cytosine on graphene using van der Waals-corrected DFT and nonequilibrium Green’s function (NEGF) methods. The four nucleobases exhibited physisorption, with calculated PBE + vdW binding energies between 0.55 and 0.74 eV and equilibrium adsorption heights of approximately 3.16–3.22 Å. The relatively strong adsorption was attributed primarily to π–π interactions between the aromatic nucleobases and the graphene π-conjugated surface. This study provides a recent theoretical reference demonstrating the relevance of graphene as an adsorptive platform for aromatic biomolecules and supports the investigation of structurally related organic molecules interacting with graphene-like carbon surfaces.

The use of finite graphene-like clusters, however, requires a careful consideration of finite-size effects. Katin et al.^25^ investigated the chemisorption of hydrogen atoms and hydroxyl groups on graphene using finite graphene clusters and demonstrated a substantial dependence of the calculated chemisorption energies on cluster size, with considerably larger clusters required to approach convergence for the systems investigated. This result highlights an important limitation of finite-cluster models: properties associated with extended graphene cannot, in general, be assumed to be independent of the cluster size. At the same time, the system investigated by Katin et al. concerns chemisorption of atomic species and therefore involves a different interaction regime from the predominantly noncovalent adsorption of the aromatic phenylpropanoids considered here. Accordingly, the cluster size behavior reported for hydrogen chemisorption should not be directly extrapolated to the present molecular physisorption problem. Nevertheless, it provides an important caution that the finite nature of the carbon model must be explicitly recognized when interpreting the adsorption energies.

Among finite PAHs, coronene (C_24_H_12_) is a compact molecular system with a high hexagonal symmetry (D6h) composed of seven fused benzene rings and characterized by an extended delocalized π-electron framework. Coronene has consequently been employed as a finite molecular representation of graphene-like carbon surfaces in theoretical investigations of molecular adsorption and electronic interactions.^26,27^ In the present work, coronene is used as a simplified finite molecular model of a local graphene-like π-conjugated surface, rather than as an exact representation of an infinite periodic graphene sheet. This distinction is important because coronene contains hydrogen-terminated edges and exhibits finite-size effects that are absent in extended graphene. Its use is therefore motivated by the objective of characterizing local adsorbate–π-system interactions while avoiding periodic boundary conditions and the computational cost associated with a much larger periodic surface.

The choice of coronene should consequently be interpreted within the scope of the present investigation. The model is intended to describe the local molecular environment in which the aromatic portion of eugenol or methyleugenol interacts with a delocalized carbon π-system. Because the principal objective is a comparative analysis of two closely related molecules within the same theoretical model, the use of an identical finite carbon framework provides a consistent basis for evaluating the effect of methylation of the phenolic hydroxyl group on adsorption. However, the model does not establish quantitative convergence to the infinite-graphene limit, and finite-size effects and edge termination remain intrinsic limitations of the approach. The adsorption results are therefore interpreted as describing interactions with a finite graphene-like carbon surface rather than as direct quantitative predictions for an infinite graphene sheet.

A further consideration is the geometry of the optimized finite clusters. Unlike an ideal periodic graphene sheet, an isolated finite PAH is a molecular system whose optimized geometry is determined by its own potential energy surface. Consequently, any small deviation from ideal graphene-like planarity in the optimized coronene structure must be regarded as a property of the finite molecular model rather than as a structural feature of an infinite graphene. For this reason, the present study does not assume that coronene reproduces all of the structural and electronic properties of extended graphene. Instead, the analysis focuses on adsorption configurations and local interaction descriptors that can be consistently evaluated for the finite-carbon model.

A comprehensive description of adsorption requires consideration of both structural and electronic characteristics of the adsorbate–surface complex. The adsorption energy (E_Ads_) provides a direct measure of the thermodynamic stability of the interaction, whereas the equilibrium adsorption distance provides information about the spatial arrangement and the nature of the adsorbate–surface interaction. Changes in the molecular electrostatic potential (MEP) can identify regions of enhanced electrophilic or nucleophilic character and help rationalize the preferred adsorption configurations. Similarly, charge-transfer analysis provides insights into the redistribution of electronic density upon adsorption and can be used to distinguish weak physisorption from interactions involving more significant electronic coupling.

Electronic structure descriptors are also particularly relevant for graphene-based sensing applications. The frontier molecular orbitals (HOMO and LUMO) and the HOMO–LUMO energy gap can provide information about changes in molecular electronic structure and chemical reactivity induced by adsorption. In addition, the density of states (DOS) and projected density of states (PDOS) enable the electronic states of the adsorbate and carbon surface to be examined separately, allowing possible changes in electronic coupling and orbital contributions associated with adsorption to be identified. Vibrational frequency calculations provide a complementary criterion for verifying that the optimized complexes correspond to local minima on the potential-energy surface and for identifying changes in vibrational characteristics associated with adsorption. Thus, the combined analysis of energetic, structural, electrostatic, electronic, charge-transfer, and vibrational descriptors provides a more comprehensive characterization of phenylpropanoid adsorption than consideration of adsorption energy alone.

Although the adsorption of organic molecules on graphene and graphene-based materials has been extensively investigated,^4,5^ and eugenol and related phenylpropanoids have been widely studied in the context of biological activity, antioxidant behavior, and corrosion inhibition,^16–23^ a systematic first-principles comparison of eugenol and methyleugenol interacting with a well-defined finite graphene-like surface remains insufficiently explored. In particular, the effect of methylation of the phenolic hydroxyl group on *E*_ads_, equilibrium geometry, electrostatic potential, charge redistribution, frontier orbital energies, DOS, and vibrational characteristics has not been systematically established within a common theoretical framework. This comparison is scientifically relevant because eugenol and methyleugenol have closely related molecular structures but differ in their hydrogen-bonding capability and electronic characteristics, thereby providing a suitable molecular pair for investigating the role of functionalization in adsorption on π-conjugated carbon surfaces.

In this work, we perform a comparative first-principles investigation of the adsorption of eugenol (C_10_H_12_O_2_) and methyleugenol (C_11_H_14_O_2_) on a coronene (C_24_H_12_) surface using DFT. Coronene is employed as a simplified finite molecular model of a local graphene-like π-conjugated surface, with the explicit recognition that finite-size effects, edge termination, and any deviation from the idealized structure of extended graphene limit direct quantitative extrapolation to infinite graphene. Within this consistent model, preferred adsorption configurations are characterized through E_Ads_ and equilibrium adsorption distances. The electronic and electrostatic characteristics of the complexes are further investigated through MEP, charge-transfer analysis, HOMO and LUMO energies, HOMO–LUMO gaps, and DOS and PDOS analyses. Vibrational frequency calculations are also employed to verify the nature of the optimized structures and to identify changes in vibrational characteristics associated with adsorption. Particular attention is given to the influence of methylation of the phenolic hydroxyl group on the interaction between the phenylpropanoid molecules and the finite π-conjugated carbon surface.

The comparative analysis of eugenol and methyleugenol is expected to clarify how the conversion of the phenolic hydroxyl group into a methoxy group affects adsorption strength, molecular orientation, charge redistribution, and electronic coupling with the coronene model. Beyond providing fundamental information on phenylpropanoid–carbon interactions, the results may contribute to the molecular understanding of graphene-based adsorption and chemical sensing systems and provide theoretical insights into the design of functional graphene-containing materials involving natural organic molecules.

The remainder of this Perspective is organized as follows. The next section describes the computational methodology employed in DFT calculations. The structural and energetic properties of the optimized adsorption complexes are then presented, followed by analyses of their electrostatic, electronic, charge transfer, and vibrational characteristics. Finally, the main conclusions of this study are summarized.

## 2 Computational Methodology

All geometries were fully optimized within the framework of DFT using the tuned long-range-corrected ωB97X-D3 exchange–correlation functional and the 6–31G­(d) basis set, i.e., *DFT/*ω*B97X-D3/6*–*31G­(d)*, as implemented in the ORCA package. This functional generally provides reliable interaction energies, HOMO–LUMO gaps, and electronic properties for molecular systems and noncovalent interactions, outperforming conventional GGA functionals, which often underestimate electronic band gaps. The D3 dispersion correction was included because the adsorption of eugenol and methyleugenol on coronene is dominated by van der Waals interactions, which play a crucial role in physisorption. The inclusion of dispersion corrections is therefore essential for accurately describing adsorption energies and equilibrium geometries in molecular adsorption systems.^28–34^

As ORCA^33,34^ is a molecular, nonperiodic code, graphene must be represented by a finite fragment. A simplified finite molecular model of a local graphene-like π-conjugated surface is represent by coronene (C_24_H_12_) with hydrogen-passivated edges, which provides a good balance between computational cost and accuracy. The optimized structures are visualized using the three-dimensional visualization programs AVOGRADO^35^ and Visual molecular dynamics (VMD).^36^

The three systems (Coronene, Eugenol, and Methyleugenol) were first optimized separately. To construct the adsorption complexes, the molecules were initially positioned parallel to the coronene plane at a distance of 3.0–3.5 Å from the surface.

Three adsorption configurations can be considered: (i) a parallel configuration (π–π stacking), in which the aromatic ring of eugenol or methyleugenol is oriented parallel to the coronene surface; (ii) a tilted configuration, where the hydroxyl group of eugenol is directed toward the coronene sheet; and (iii) a vertical configuration, with the molecule oriented approximately perpendicular to the coronene plane.

All adsorption geometries were fully optimized using the (i) parallel adsorption configuration (π–π stacking), and their total energies (*E*_*Total*_ = *E*_*Complex*_) were compared to identify the most stable adsorption configuration. The adsorption energy (*E*_*Ads*_) or binding energy of eugenol/methyleugenol on coronene was calculated by eq 1

1 $$E_{Ads}=\left[E_{Total}-\left(E_{Coronene}+E_{Molecule}\right)\right]$$

where *E*_*Total=*_E_Complex_ is the total energy of the adsorbed system or formed complex, *E*_*Coronene*_ is the energy of the isolated coronene model, and *E*_*Molecule*_ is the energy of the isolated molecule. Negative *E*_*Ads*_ values indicate an energetically favorable interaction and support the formation of stable adsorbate–surface complexes within the electronic structure framework. However, because thermal, zero-point-energy, and entropic contributions were not explicitly included, E_Ads_ alone should not be interpreted as a direct measure of thermodynamic favorability in terms of the Gibbs free energy.

Optimized structures with bond length, single-point and adsorption energies, charge transfer, density of states (DOS), frontier molecular orbitals (HOMO, LUMO, and E_gap_ = E_LUMO_–E_HOMO_), molecular electrostatic potential (MEP) map, global reactivity parameters, infrared (absorbance and transmittance), and Raman spectra were subsequently analyzed to evaluate the interaction mechanisms between the adsorbates and coronene.

Global reactivity parameters (GRPs) are electronic and thermodynamic descriptors derived from DFT calculations. Based on the energies of the frontier molecular orbitals (HOMO and LUMO), these parameters provide valuable information about the chemical reactivity, stability, and electron-donating or electron-accepting ability of a neutral form. The quantum molecular descriptors (QMDs) of the structures are determined as follows by (eqs 2–9):

2 $$E_{gap}=E_{LUMO}-E_{HOMO}$$

3 $$EA\approx -E_{LUMO}$$

4 $$IP\approx -E_{HOMO}$$

5 $$\mu =-\left(IP+EA\right)/2\approx \left(E_{LUMO}{+E}_{HOMO}\right)/2$$

6 $$\chi =-\mu \approx -\left(E_{LUMO}{+E}_{HOMO}\right)/2$$

7 $$\eta =\left(IP-EA\right)/2\approx \left(E_{LUMO}{-E}_{HOMO}\right)/2$$

8 $$S=1/2\eta \approx 1/\left(E_{LUMO}{-E}_{HOMO}\right)$$

9 $$\omega =\mu^{2}S\approx {\left(E_{LUMO}{+E}_{HOMO}\right)}^{2}/4.\left(E_{LUMO}{-E}_{HOMO}\right)$$

10 $${\Delta N}_{\max}=-\mu /\eta \approx -\left(E_{LUMO}{+E}_{HOMO}\right)/\left(E_{LUMO}{-E}_{HOMO}\right)$$

where *EA* is the electron affinity, *IP* is the ionization potential or energy, μ is the chemical potential, χ is the electronegativity, η is the chemical hardness, *S* is the global softness, ω is the electrophilicity index, and *ΔN*_*max*_ is the minimum fraction of transferred electron. eqs 3 and ([Disp-formula eq4]) were estimated using the conventional Koopmans-type relationships, *IP* ≈ –*E*_*HOMO*_ and *EA* ≈ –*E*_*LUMO*_, respectively. These quantities should be regarded as approximate estimates derived from the Kohn–Sham orbital energies from DFT calculations. In exact DFT, the relationship between the ground-state frontier molecular orbital energies and the IP and EA involves the derivative discontinuity (-Δ_XC_). Thus, the present methodology employs Koopmans-type approximations as a computationally inexpensive approach for estimating these energetic properties without performing separate, computationally demanding calculations for the ionized or electron-added states.^37–41^

## 3 Results and Discussion

### 3.1 Optimized Adsorption Geometries

Geometry optimizations of the bio-organic molecules, coronene, and their complexes (1 and 2) were performed using the ORCA package for neutral molecules in their singlet ground electronic state, see Figure 1(a–e). In the present study, we investigated four distinct adsorption positions for each system and identified the lowest energy structure among the configurations considered. For Complexes 1 and 2, the corresponding lowest energy configurations, together with three additional local-minimum configurations (extra 1, extra 2, and extra 3), are presented in Figure 1(d–e) and Tables S1 and S2 of the Supporting Information.

**Figure 1:**
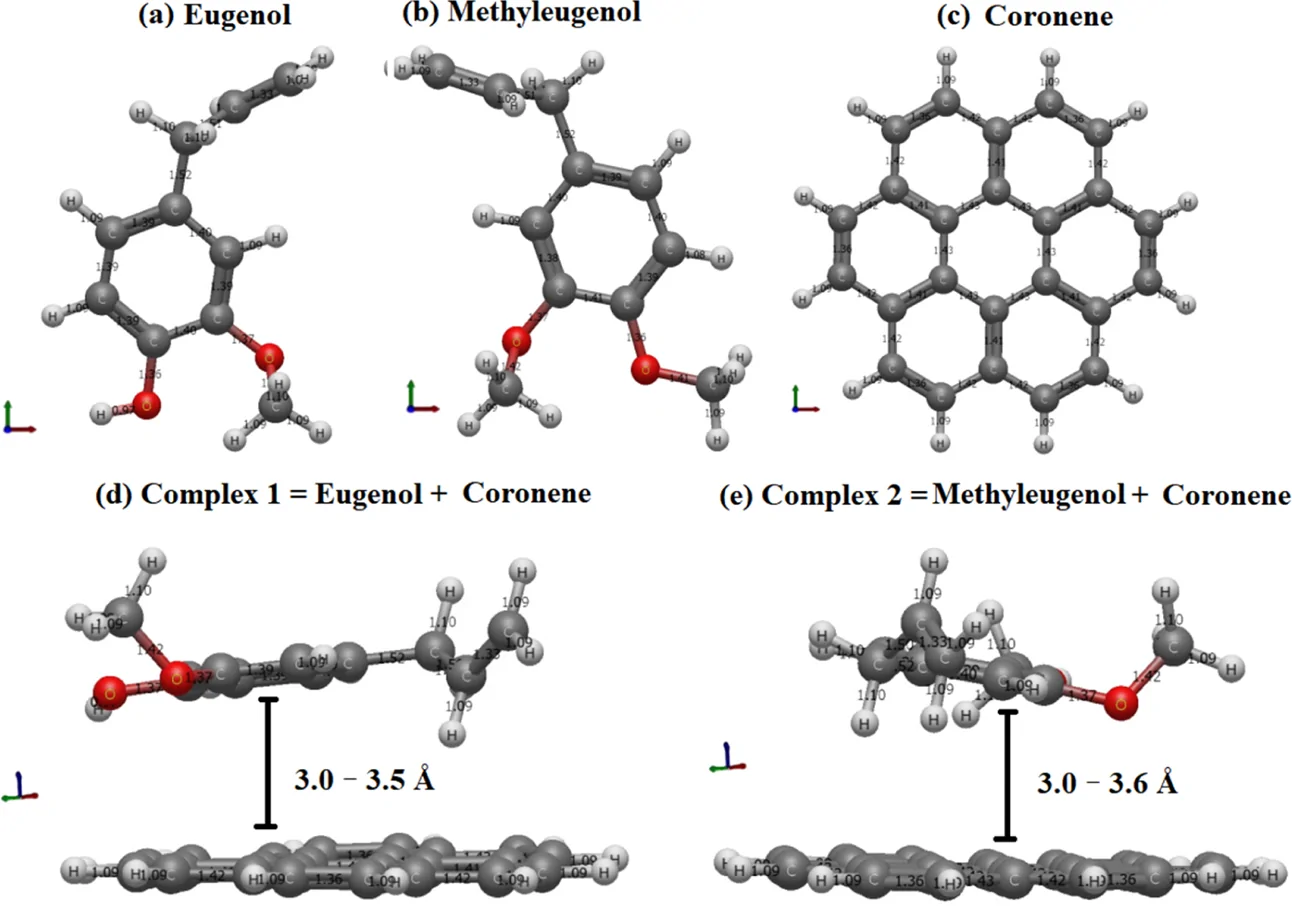
Optimized geometries and bond lengths of the bio-organic molecules [(a) eugenol (C_10_H_12_O_2_) and (b) methyleugenol (C_11_H_14_O_2_)], (c) coronene (C_24_H_12_), (d) complex 1 (eugenol + coronene), and (e) complex 2 (methyleugenol + coronene) at their lowest energy structures. The Cartesian coordinate axes are shown in red (x), green (y), and blue (z). Carbon (C), hydrogen (H), and oxygen (O) atoms are represented by gray, white, and red spheres, respectively.

The corresponding total energies were −1460.1760 E_h_ for complex 1 (eugenol + coronene) and −1499.4714 E_h_ for complex 2 (methyleugenol + coronene). Considering that 1 hartree (E_h_) = 27.2114 eV, these energies correspond to −39 733.6726 eV and −40 802.9636 eV, respectively.

Because the present calculations did not involve an exhaustive exploration of all possible adsorption sites, molecular orientations, and conformations, these structures are termed here as the lowest energy configurations among those investigated rather than as absolute global minima. Establishing the true global minimum would require a systematic exploration of a substantially larger configuration space.

The dimensions of the three systems are as follows: eugenol has a length of 8.307 Å, methyleugenol has a length of 9.977 Å, and coronene has a length of 9.231 Å, a width of 9.224 Å, and a projected area (length × width) of ≈85.146 Å^2^. Figure 1a shows that, for eugenol, the aromatic-ring C–H, C=C, and C–C bond lengths are 1.09, 1.39, and 1.40 Å, respectively. In the allyl chain (H_2_C=CH–CH_2_), the C=C bond length is 1.33 Å, while the C–H bond lengths are 1.10 and 1.09 Å. The C–C bond lengths between the aromatic ring and the CH_2_ group are 1.52 and 1.51 Å. The C–O bond lengths are 1.36, 1.37, and 1.42 Å, and the O–H bond length is 0.97 Å. For methyleugenol [Figure 1b], the hydroxyl group (O–H) is replaced by a methoxy group (O–H_3_C), resulting in C–O and C–H bond lengths of 1.41 instead of the O–H bond and 1.10 Å, respectively. Figure 1c presents the optimized bond lengths of coronene. In the central region, the C=C and C–C bond lengths are 1.41 and 1.43 Å, respectively, whereas in the edge region they are 1.36 and 1.42 Å, respectively. The C–H bond lengths are 1.09 Å.

Both eugenol and methyleugenol preferentially adsorb in a parallel orientation relative to the coronene surface, maximizing π–π stacking interactions between the aromatic ring and the carbon lattice, see Figure 1(d,e). The equilibrium distances between the eugenol and methyleugenol molecules and coronene surface are found to be in the range of 3.0–3.5 Å and 3.0–3.6 Å, characteristic of physisorption regimes.

Subsequently, the Multiwfn software^42^ was used to analyze the structural distortion of complexes 1 and 2 relative to the isolated coronene surface optimized at the *DFT/*ω*B97X-D3/6*–*31G­(d)* level. Two geometric descriptors related to molecular planarity were calculated: the molecular planarity parameter (MPP), defined as the mean deviation of the atoms from the best–fit plane, and the span of deviation from the plane (SDP), defined as the difference between the largest and smallest atomic deviations from the plane.^43^

According to Table 1, to further assess the structural changes associated with complex formation, the MPP and SDP were evaluated for coronene, complex 1 (eugenol + coronene), and complex 2 (methyleugenol + coronene).

**Table 1:** MPP and SDP (in Å) Calculated for All Carbons of Coronene, Complexes 1, and 2 Optimized at the *DFT/*ω*B97X-D3/6-31G­(d)* Level

systems	MPP (Å)	SDP (Å)
coronene	0.000006	0.000021
complex 1 (eugenol + coronene)	0.036450	0.118431
complex 2 (methyleugenol + coronene)	0.029002	0.097955

Coronene exhibits extremely small MPP and SDP values of 0.000006 and 0.000021 Å, respectively, confirming its essentially planar geometry. Upon complex formation, slightly larger deviations from planarity are observed for both bio-organic molecules. Complex 1 (eugenol + coronene) presents MPP and SDP values of 0.036450 and 0.118431 Å, respectively, whereas the corresponding values for complex 2 (methyleugenol + coronene) are 0.029002 and 0.097955 Å. These results indicate that both adsorbed molecules retain a high degree of planarity, although their interaction with the coronene surface induces a small structural distortion. Notably, the lower MPP and SDP values obtained for complex 2 (methyleugenol + coronene) indicate a slightly smaller deviation from planarity than that observed for complex 1 (eugenol + coronene). Overall, the relatively low MPP and SDP values for both complexes suggest that adsorption on the coronene surface does not substantially disrupt the molecular structures, with complex 1 (eugenol + coronene) exhibiting a somewhat greater degree of nonplanarity.

### 3.2 Adsorption Energy (*E*_*Ads*_)

For aromatic systems adsorbed on graphene, the adsorption energies (*E*_*Ads*_) typically reported in the literature are given by Table 2.

**Table 2:** Type of Interaction versus E_Ads_ for Aromatic Systems Adsorbed on Graphene^42^

type of interaction	E_Ads_ (eV)
weak physisorption (van der waals interactions)	–0.10 to −0.50
moderate physisorption	–0.50 to −1.00
chemisorption	<- 1.00

Since both eugenol and methyleugenol contain an aromatic ring capable of interacting with the π-electron system of the coronene, the adsorption process is expected to be predominantly governed by π–π stacking interactions, leading to a physisorption mechanism.

A stronger adsorption of methyleugenol relative to eugenol is generally expected due to its higher electronic polarizability, the presence of an additional methoxy group, and a greater number of π-electrons available for interactions with the graphene surface. Consequently, the following trend is typically observed *|E*_*Ads*_ *(Methyleugenol)| > |E*_*Ads*_ *(Eugenol)|* with adsorption energy differences commonly ranging from 0.02 to 0.20 eV.

Table 3 presents the calculated adsorption energies (*E*_Ads_) of eugenol, methyleugenol, and the coronene, as well as those of the adsorption complexes formed between coronene and the adsorbate molecules, namely, complex 1 (eugenol + coronene) and complex 2 (methyleugenol + coronene).

**Table 3:** Energies for Isolated Systems (E_Eugenol_, E_Methyleugenol_, and E_Coronene_), Complexes (E_Total 1_ = E_Eugenol+Coronene_, and E_Total 2_ = E_Methyleugenol+Coronene_), and *E*_*Ads*_ ^a^

*systems*	*energies (hartree - E*_*h*_ *)*	*energies (eV)*	*E*_*Ads*_ *(hartree - E*_*h*_ *)*	*E*_*Ads*_ *(eV)*
eugenol	–538.5308	–14,654.17	-	-
methyleugenol	–577.8252	–15,723.43	-	-
coronene	–921.6215	–25,078.61	-	-
complex 1 = eugenol + coronene	–1460.1760	–39,733.43	–0.0237	–0.645
complex 2 = methyleugenol + coronene	–1499.4714	–40,802.71	–0.0247	–0.672

a Consider that 1 hartree (E_h_) = 27.2114 eV.

In Table 3, the negative *E*_*Ads*_ obtained for both systems (−0.645 and −0.672 eV for complexes 1 and 2, respectively) indicate that the formation of the optimized adsorbed configurations is energetically favorable within the electronic structure framework employed. The slightly more negative value obtained for complex 2 suggests a somewhat stronger electronic stabilization of methyleugenol on the coronene surface relative to eugenol. However, these electronic E_Ads_ should not be interpreted as direct evidence of thermodynamic favorability, since thermal, entropic, zero-point-energy, and other contributions to the Gibbs free energy were not comprehensively evaluated in the present study. Therefore, the present results describe the electronic energetic preference of the calculated configurations rather than their complete thermodynamic stability under experimental conditions.

The calculated *E*_*Ads*_ were found to be −0.645 eV for eugenol/coronene and −0.672 eV for methyleugenol/coronene, yielding a difference of 0.027 eV. Although smaller than the typical range reported in the literature, this result still indicates a slightly weaker interaction of methyleugenol with the coronene. The *E*_*Ads*_ in the range of −0.645 eV and −0.672 eV are typical of moderate physisorption, see Table 2, dominated by π–π interactions and van der Waals dispersion forces. These results suggest that coronene (C_24_H_12_) has potential to act as a molecular recognition platform or sensor for phenolic compounds derived from eugenol.

As presented in Table 3, the negative *E*_*Ads*_ and the predominantly dispersive character of the interaction indicate favorable electronic interaction between the phenylpropanoid molecules and the graphene-like carbon model. However, the calculated *E*_Ads_ alone does not establish thermodynamic stability in terms of Gibbs free energy or demonstrate corrosion inhibition performance under experimental conditions. The present results therefore provide a molecular-level basis for understanding the interaction of eugenol and methyl–eugenol with graphene-like carbon surfaces and may be relevant to future investigations of these compounds in graphene-based sensing and corrosion protection systems.

The calculated adsorption behavior provides molecular-level insights into the interaction of eugenol and methyl eugenol with a graphene-like carbon surface, which may be relevant to their potential role as corrosion inhibitor molecules. The relatively favorable electronic interaction and predominance of dispersion contributions suggest that these molecules can interact noncovalently with the modeled carbon surface. However, the present calculations do not directly establish the corrosion inhibition efficiency. In realistic corrosion environments, additional factors such as metallic substrates, solvents, electrolyte composition, temperature, competing adsorbates, surface defects, and electrochemical conditions can substantially influence the adsorption and inhibition behavior. Therefore, the present results should be regarded as a molecular-level computational assessment that may motivate further theoretical and experimental investigations of these compounds in realistic corrosion systems.

The interaction of eugenol and methyleugenol with the coronene model also provides molecular-level information that may be relevant to future investigations of carbon-based sensing systems. Changes in the electronic structure, charge redistribution, and adsorption energetics upon molecular binding can provide useful descriptors for exploring possible sensing mechanisms. Nevertheless, the present calculations do not directly simulate sensor operation or quantify the sensing performance. Realistic sensing platforms involve extended surfaces, substrates, defects, solvent effects, competing adsorbates, temperature, and other environmental factors that are not represented in the isolated coronene model. Thus, the present results should be interpreted as fundamental molecular-level insights rather than as direct evidence of the sensing performance.

In Tables S3 to S6, we performed an Energy Decomposition Analysis (EDA) and EDA-Natural Orbitals for Chemical Valence (EDA-NOCV) methods^44^ at DFT/wB97X-D3 6–31G­(d) to provide a deeper insight into the nature of the interaction between eugenol/methyleugenol and the coronene surface. The EDA provides a quantitative assessment of the relative contributions of electrostatic (E_Elet_), orbital/polarization (E_Orb_), dispersion (E_Disp_), Pauli repulsion (E_Pauli_), and exchange–correlation (E^0^_XC_) interactions to the overall interaction energy (E_Bond_), as well as to characterize the associated charge-transfer contributions through the NOCV channels, see Figures S1 and S2. These results further support the predominantly physisorption-driven character of the adsorption process, as the dispersion contribution is the dominant stabilizing component, followed by the electrostatic and orbital contributions (|E_disp_| > |E_elet_| > |E_orb_|).

The EDA results provide quantitative evidence for the nature of the interactions between the bio-organic molecules (eugenol and methyleugenol) and the coronene surface. For complex 1, the dispersion contribution E_Disp_ = −12.90 kcal/mol is the largest attractive component, followed by the electrostatic E_Elet_ = −6.14 kcal/mol and orbital E_Orb_ = −2.38 kcal/mol contributions. A similar trend is observed for complex 2, for which the dispersion contribution is −14.17 kcal/mol, compared with −5.06 and −2.55 kcal/mol for the electrostatic and orbital contributions, respectively. Thus, dispersion represents the predominant attractive contribution in both complexes, consistent with the expected role of noncovalent van der Waals interactions between the aromatic surface and the bio-organic molecules. Nevertheless, the substantial Pauli repulsion (+43.63 and +46.76 kcal/mol for complexes 1 and 2, respectively) partially offsets the attractive interactions, highlighting the balance between attractive contributions, including *E*_XC_^0^, and repulsive contribution in determining the final interaction energy (E_Bond_).

The EDA-NOCV analysis further indicates relatively modest orbital contributions compared with the dispersion contribution obtained from the EDA. The five most significant NOCV channels yield total interaction-energy contributions (∑Δ*E*_*k*_) of approximately −1.44 and −1.40 kcal/mol for complexes 1 and 2, respectively. Furthermore, the values of ∑Δ*T*_*k*_ are positive, whereas the potential components, ∑Δ*V*_*k*_, are negative and of greater magnitude, resulting in small negative values of ∑Δ*E*_*k*_. This behavior is consistent with the net stabilization associated with the interaction channels analyzed. The corresponding density-deformation channels reveal localized electron-density redistribution upon complex formation. However, because the net charge transfer is relatively small, these density rearrangements should be interpreted as complementary signatures of intermolecular electronic interaction rather than evidence of a dominant charge-transfer mechanism. Overall, the EDA-NOCV results support a predominantly noncovalent interaction picture in which dispersion provides the major attractive contribution, while electrostatic and orbital interactions provide additional, smaller contributions.

#### 3.2.1 BSSE Correction for Adsorption Energy (*E*_*Ads*_) Calculated with Different Functionals and Basis Sets

Given the relatively small size of the molecular system investigated, it is entirely feasible and highly desirable to compare different exchange–correlation functionals and basis sets, thereby providing a more reliable assessment of the computational methodology. Although 6–31G­(d) is an acceptable starting point, the reliability of the conclusions would be significantly strengthened by evaluating larger basis sets, including diffuse functions. Additional benchmark calculations were performed to evaluate the influence of both the exchange–correlation functional and the basis set on the calculated adsorption properties. In addition to the original ωB97X-D3/6–31G­(d) methodology, calculations were performed using ωB97X-D3/def2-TZVPD, B3LYP-D3­(BJ)/6–31G­(d), and B3LYP-D3­(BJ)/def2-TZVPD. Because weak molecular interactions are particularly susceptible to basis set superposition error (BSSE), BSSE corrections were applied at all levels of theory using the Boys–Bernardi counterpoise (BB-CP) method implemented in the ORCA package.

The def2-TZVPD basis set is considered a more modern alternative to 6–311++G­(d,p), as it provides a triple-ζ description of the valence region together with diffuse functions, allowing a more accurate representation of the electron density and noncovalent interactions. This basis set is also particularly suitable for DFT-based adsorption calculations performed with quantum chemistry packages such as ORCA. Furthermore, the B3LYP-D3­(BJ) functional was considered because it includes the Grimme D3­(BJ) dispersion correction, which improves a more appropriate description of long-range dispersion interactions and the accuracy of noncovalent interaction energies, which are essential for adsorption processes involving π-conjugated systems.

The BSSE-corrected adsorption energy of eugenol/methyleugenol on coronene was calculated using BB-CP methot, as given by eq 11,^45,46^ see Tables S7 and S8

11 $$E_{Ads-BB-CP}=E_{Ads}-\delta E_{BSSE=}\left[E_{Total}-\left(E_{Coronene}+E_{Molecule}\right)\right]-\left[E_{GhostCoronene}-E_{DeformedCoronene}+E_{GhostMolecule}-E_{DeformedMolecule}\right]$$

where: *E*_*Ads‑BB‑CP*_ is Boys–Bernardi counterpoise-corrected adsorption energy which is obtained from calculations using ghost functions, *E*_*Ads*_ is the uncorrected adsorption energy given by eq 1, δ*E*_*BSSE*_ is the BSSE correction applied to *E*_*Ads*_ to obtain *E*_*Ads‑BB‑CP*_, *E*_*Ghost Coronene*_ and *E*_*Ghost Molecule*_ are the energies of the isolated molecules with the geometry of the complex system used in ghost atoms to add a set of basis functions to another molecule, and *E*_*Deformed Coronene*_ and *E*_*Deformed Molecule*_ are the energies of the isolated molecules with a geometry found within the complex.

Table 4 presents the calculated *E*_*Ads*_, δ*E*_*BSSE*_, and *E*_*Ads‑BB‑CP*_ for the two complex systems with DFT/ωB97X-D3/6–31G­(d), DFT/ωB97X-D3/def2-TZVPD, DFT/B3LYP-D3­(BJ)/6–31G­(d), and DFT/B3LYP-D3­(BJ)/def2-TZVPD methodologies. As expected in the calculations, the δ*E*_*BSSE*_ (6–31G­(d)) > δ*E*_*BSSE*_ (*def2-TZVPD*). This occurs because def2-TZVPD is a much more flexible basis set and includes diffuse functions, allowing for a more adequate electronic description of the interaction between the molecule and the coronene surface. Since eugenol and methyleugenol interact with coronene primarily through dispersion and other noncovalent interactions, BSSE is particularly relevant to the adsorption energies. However, it is important to assess the extent to which the δ*E*_*BSSE*_ correction affects *E*_*Ads*_, by evaluating the δ*E*_*BSSE*_/*E*_*Ads*_ ratio. The results indicate that the def2-TZVPD basis set provides more reliable adsorption energies, as it exhibits a lower relative BSSE contribution.

**Table 4:** E_Ads_ Calculations for the Two Complex Systems Using the DFT/ωB97X-D3/6-31G­(d), DFT/ωB97X-D3/def2-TZVPD, DFT/B3LYP-D3­(BJ)/6-31G­(d), and DFT/B3LYP-D3­(BJ)/def2-TZVPD Methodologies^a^

*energies (systems)/method*	*wB97X-D*3/6*–31G(d)*	*wB97X-D3/def2-TZVPD*	*B3LYP D3BJ/6–31G(d)*	*B3LYP D3BJ/def2-TZVPD*
*E*_*Ads*_ (complex 1 = eugenol + coronene)	–0.642 eV	–0.563 eV	–0.740 eV	–0.606 eV
δ*E*_*BSSE*_ (complex 1 = eugenol + coronene)	0.185 eV	0.027 eV	0.225 eV	0.029 eV
*E*_*Ads‑BB‑CP*_ (complex 1 = eugenol + coronene)	–0.457 eV	–0.536 eV	–0.515 eV	–0.577
δ*E*_*BSSE*_ */E*_*Ads*_ (complex 1 = eugenol + coronene)	28.8%	4.79%	30.4%	4.78%
*E*_*Ads*_ (complex 2 = methyleugenol + coronene)	–0.669 eV	–0.574 eV	–0.772 eV	–0.625 eV
δ*E*_*BSSE*_ (complex 2 = methyleugenol + coronene)	0.206 eV	0.0299 eV	0.253 eV	0.032 eV
*E*_*Ads‑BB‑CP*_ (complex 2 = methyleugenol + coronene)	–0.463 eV	–0.544 eV	–0.519 eV	–0.593 eV
δ*E*_*BSSE*_ */E*_*Ads*_ (complex 2 = methyleugenol + coronene)	30.8%	5.2%	32.7%	5.12%

a Consider that 1 hartree (E_h_) = 27.2114 eV.

The BSSE analysis revealed a pronounced basis-set dependence of the calculated adsorption energies. For both eugenol/coronene and methyleugenol/coronene complexes, the relative BSSE contribution was approximately 29–33% when the 6–31G­(d) basis set was employed, indicating a substantial artificial stabilization associated with basis-set incompleteness. In contrast, increasing the basis-set size to def2-TZVPD markedly reduced the BSSE contribution to approximately 4.8–5.2%, irrespective of the density functional used. These results indicate that the def2-TZVPD basis set provides a substantially more reliable description of the adsorption interactions, with a much lower sensitivity to basis-set superposition effects.

The relatively large BSSE contribution obtained with the 6–31G­(d) basis set indicates that the uncorrected adsorption energies at this level may overestimate the interaction strength and should therefore not be considered as the primary quantitative reference. Accordingly, the counterpoise-corrected adsorption energies obtained with the larger def2-TZVPD basis set are emphasized as the preferred quantitative values in this study.

A slightly larger BSSE contribution was observed for methyl eugenol than for eugenol for all levels of theory. The calculated difference between the E_Ads_ values of eugenol and methyleugenol is indeed small, particularly when the larger basis set and BSSE correction are considered. We describe the results as indicating a small computational trend toward a somewhat stronger interaction for methyleugenol within the present model and computational methodology.

The optimized geometries, adsorption energies, and key electronic descriptors obtained with the different computational protocols were compared to those from the original methodology. The benchmark calculations showed only minor quantitative differences, while the relative stabilities of the adsorption configurations and the main conclusions remained unchanged. These results confirm that the computational findings are robust with respect to the choice of the functional and basis set.

Deka, Saikia, and Srivastava^47^ demonstrated that monoterpenoids (such as arvacrol, carvone, γ-terpinene, limonene, and menthol) absorbs more efficiently on the surface of nitrogen-doped graphene compared to graphene using the DFT calculation carried out in water medium at B3LYPX/6–31G­(d) and M06–2XX/6–31G­(d) levels. This study offers an effective way to design promising adsorbent for selective separation of the terpenoids.

#### 3.2.2 The Influence of Finite Size and Edge Effects

Finite size and edge effects were taken into account by comparing the non-hydrogenated graphene model (C_24_) with the coronene-based finite model (C_24_H_12_). Unlike an infinite graphene sheet, the coronene molecule contains a finite number of carbon atoms and hydrogen-terminated edges, which can introduce localized electronic states, alter the distribution of charge density, and affect the frontier molecular orbitals (HOMO and LUMO). These edge atoms can also rise to finite-size effects that are absent in an ideal periodic or non-hydrogenated graphene sheet. In particular, previous studies have shown that electronic structures in finite coronene-based graphene nanoflakes can be significantly influenced by the molecular edges.^48–51^

In the present study, coronene (C_24_H_12_) was employed as a finite molecular model of graphene, although it does not reproduce all the properties of an extended graphene sheet. The main advantage of this model is that it preserves the characteristic sp^2^-hybridized carbon framework and extended π-conjugated system of graphene while allowing the adsorption process to be investigated using DFT calculations. However, unlike an infinite periodic or non-hydrogenated graphene sheet, coronene contains hydrogen-terminated edges. These edge atoms may introduce finite-size effects and modify the electronic structure, particularly the distribution of frontier molecular orbitals and local charge density.^48–50^

Previous computational studies have demonstrated that coronene is a commonly used finite model for graphene and that increasing the size of the graphene-like model can affect the magnitude of E_Ads_. Nevertheless, the overall adsorption behavior can remain qualitatively consistent, while the coronene model offers a substantially lower computational cost.^48^ Thus, the calculated adsorption energies should be interpreted as a characteristic of the finite coronene model rather than as exact values for an infinite or nonhydrogenated graphene sheet.

For the present systems, the *E*_*Ads‑BB‑CP*_ of −0.457 eV for complex 1 (eugenol + coronene) and −0.463 eV for complex 2 (methyleugenol + coronene) calculated by *DFT/*ω*B97X-D3/6*–*31G­(d)* indicate relatively weak-to-moderate interactions between the adsorbates and the coronene surface, see Table 4. The optimized structures show that the aromatic rings of eugenol and methyleugenol remain close to the π-conjugated carbon surface, supporting the interpretation of the interaction predominantly as physisorption involving π–π interactions and dispersion forces. Importantly, the difference between the *E*_*Ads‑BB‑CP*_ of the two complexes is equal to 0.006 eV, indicating that the main conclusions concerning their comparative adsorption behavior are not expected to depend critically on the precise magnitude of the finite-size effect.

We have performed additional *DFT/*ω*B97X-D3/6*–*31G­(d)* calculations for the *E*_*Ads‑BB‑CP*_ and equilibrium geometries of eugenol and methyleugenol interacting with a non-hydrogenated C_24_ graphene-like carbon cluster. These additional calculations are provided in Tables S9 and S10 of the Supporting Information. Among the configurations investigated, the lowest energy configurations yielded E_Ads‑BB‑CP_ values of −0.475 eV for complex 3 (eugenol + non-hydrogenated C_24_) and −0.464 eV for complex 4 (methyleugenol + non-hydrogenated C_24_), calculated at the DFT/ωB97X-D3/6–31G­(d) level. The difference between these values is only 0.011 eV, indicating very similar adsorption strengths.

The comparison with the hydrogen-terminated coronene model shows that the E_Ads‑BB‑CP_ values are relatively close for the two C_24_-based (nonhydrogenated C_24_ graphene-like) models. This result indicates that the principal features of the adsorption interaction are reasonably consistent between the hydrogen-terminated and nonhydrogenated finite carbon models, while also revealing the quantitative differences associated with edge termination and finite-size effects. Therefore, the additional benchmark provides useful support for the use of coronene as a finite graphene-like model without implying quantitative equivalence with an infinite periodic graphene sheet.

Table 5 presents the calculated *E*_*Ads*_, δ*E*_*BSSE*_, *E*_*Ads‑BB‑CP*_, and δ*E*_*BSSE*_ */E*_*Ads*_ values for the two complex systems obtained using the DFT/ωB97X-D3/6–31G­(d) methodology. As expected, the calculated δ*E*_*BSSE*_ values for coronene complexes 1 and 2 using the 6–31G­(d) basis sets, 0.185 and 0.206 eV, respectively, are very close to those obtained for non-hydrogenated graphene complexes 3 and 4, 0.200, and 0.195 eV, respectively. The close agreement between δ*E*_*BSSE*_ for the coronene and graphene models indicates that the basis-set-related effects are comparable in both systems. This consistency supports the use of the finite coronene model as a reasonable computational representation for assessing the adsorption interactions, while recognizing that differences in the absolute E_Ads_ may still arise from the finite size and edge effects inherent to the coronene model.

**Table 5:** E_Ads_ Calculations for the Two New Complex Systems Using the DFT/ωB97X-D3/6-31G­(d) Methodology^a^

*energies (systems)/method*	*wB97X-D*3/6*–31G(d)*	*energies (systems)/method*	*wB97X-D*3/6*–31G(d)*
*E*_*Ads*_ (complex 3 = eugenol + graphene)	–0.676 eV	*E*_*Ads*_ (complex 4 = methyleugenol + graphene)	–0.659 eV
δ*E*_*BSSE*_ (complex 3 = eugenol + graphene)	0.200 eV	δ_*EBSSE*_ (complex 4 = methyleugenol + graphene)	0.195 eV
*E*_*Ads‑BB‑CP*_ (complex 3 = eugenol + graphene)	–0.475 eV	*E*_*Ads‑BB‑CP*_ (complex 4 = methyleugenol + graphene)	–0.464 eV
δ_*EBSSE*_ */E*_*Ads*_ (complex 3 = eugenol + graphene)	29.5%	δ_*EBSSE*_ */E*_*Ads*_ (complex 4 = methyleugenol + graphene)	29.5%

a Consider that 1 hartree (E_h_) = 27.2114 eV.

The ratios δ*E*_*BSSE*_ */E*_*Ads*_ for complexes 1 and 2 are 30.8% and 32.7%, respectively, whereas a value of 29.5% is obtained for both complexes 3 and 4. These results indicate that the BSSE correction (δ*E*_*BSSE*_) represents approximately one-third of the calculated adsorption energy in all systems. Despite the slightly higher ratios observed for the coronene complexes, the values remain relatively close to those obtained for the graphene complexes, indicating that BSSE has a comparable relative contribution to the adsorption energies in the two models. This finding further supports the consistency of the computational treatment and suggests that the comparison between the finite coronene and graphene models is not strongly affected by differences in the relative magnitude of the BSSE correction. Nevertheless, the remaining differences in the adsorption energies should be interpreted in the context of the distinct structural characteristics and finite-size and edge effects of the coronene model.

### 3.3 Charge Transfer Analysis

The Löwdin population analysis method was employed to calculate atomic charges (Q_coronene_ = Q_molecule_ = Σ_i_ q_i_) and charge transfer (CT = |Q_coronene_| ≈ |Q_molecule_|) in the complexes 1 and 2. Löwdin population analysis performs a symmetric orthogonalization of the basis functions prior to the population analysis, which can make the resulting atomic charges less problematic than Mulliken charges in this case, whereas the Hirshfeld method partitions the molecular electron density according to the promolecule reference density.

In Figure 2 (a–e), the charge-transfer (CT) analysis between the adsorbate molecules (eugenol and methyleugenol) and the coronene was performed to investigate the electronic redistribution upon adsorption. A comparison of the charge distribution maps of the adsorption complexes with those of their isolated constituents reveals distinct interaction patterns between each molecule and coronene. For methyleugenol, adsorption results in only slight perturbations of the coronene charge distribution, indicating a weak interaction. In contrast, the eugenol–coronene complex shows evident charge redistribution between the two oxygen atoms of eugenol and the adjacent carbon atoms of coronene, demonstrating a stronger intermolecular interaction than that observed for methyleugenol.

**Figure 2:**
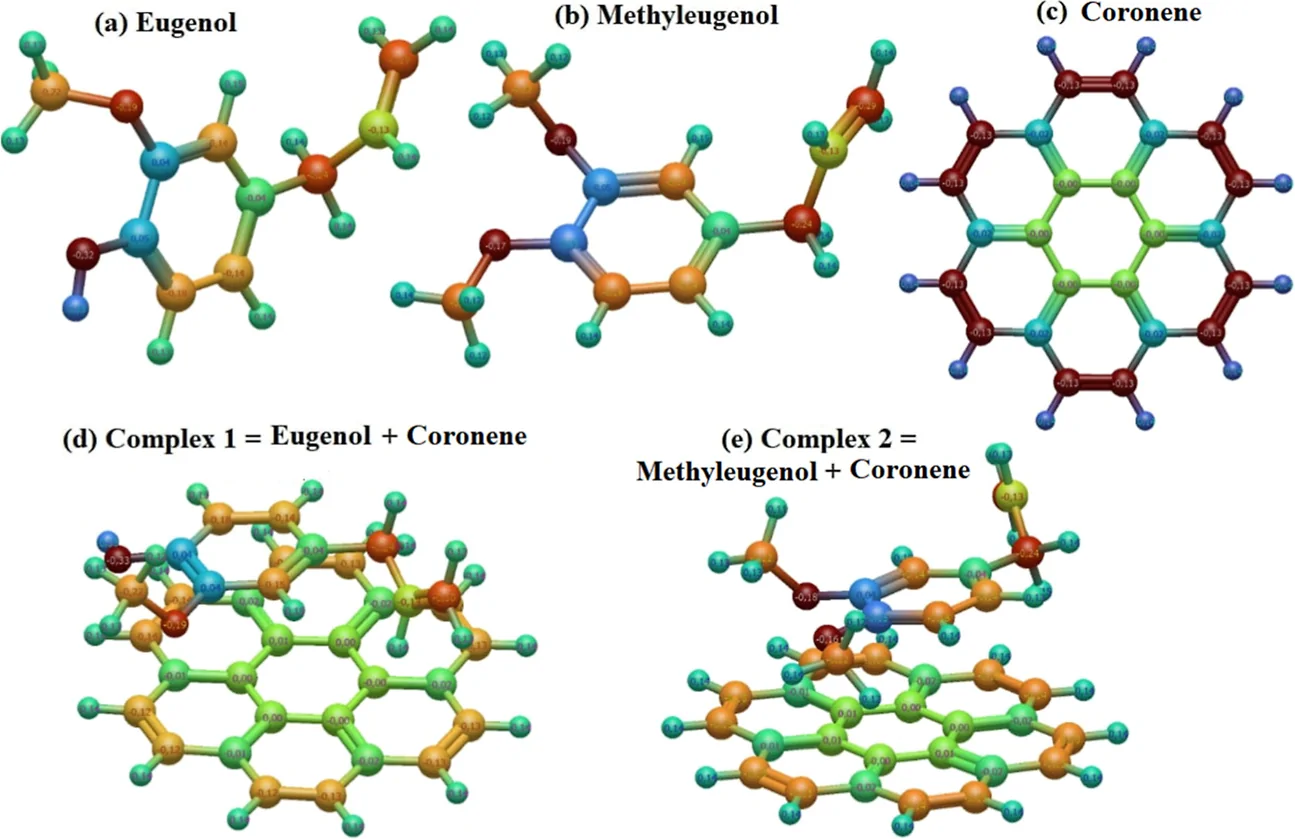
Charge-transfer analysis between the adsorbate molecules and the surface: (a) eugenol, (b) methyleugenol, (c) coronene, (d) complex 1, and (e) complex 2. The atom colors represent the calculated atomic charges: blue indicates positive charge, red indicates negative charge, and green indicates approximately zero charge. The color scale was uniformly normalized using the charge with the largest absolute value, resulting in a symmetric range.

The results indicate that a small but non-negligible charge transfer (<0.05 e) occurs from the adsorbate molecules to the coronene surface (or vice versa, depending on the calculated sign convention), confirming the presence of weak electronic coupling between the π-electron systems of the molecules and the nanoribbon.

Such charge transfer is consistent with a physisorption regime dominated by π–π stacking and van der Waals interactions. In this context, methyleugenol is expected to exhibit slightly higher charge transfer compared to eugenol due to its increased polarizability and the presence of an additional methoxy group (−OCH_3_), which enhances its electron-donating character.

Overall, the charge-transfer analysis supports the *E*_Ads_ results, indicating that both systems interact weakly but effectively with the coronene surface, which is a desirable feature for molecular sensing applications.

### 3.4 Electronic Structure

Figure 3a–e show frontier molecular orbitals (FMOs) (inset graph in Origin 8.0) and density of states (DOS) of the adsorbate molecules, the surface, and the adsorption complexes. The FMOs (HOMO and LUMO) and DOS of eugenol and methyl eugenol were analyzed with Multiwfn^42,52,53^ and VMD^36^ software to gain insights into their electronic properties and adsorption behavior on the coronene surface. The FMOs were plotted by using an isosurface value of 0.01 au. The dashed and solid vertical lines indicate E_HOMO_ and E_LUMO_, respectively, and the separation between them represents the energy gap (E_gap_) given by eq 2.

**Figure 3:**
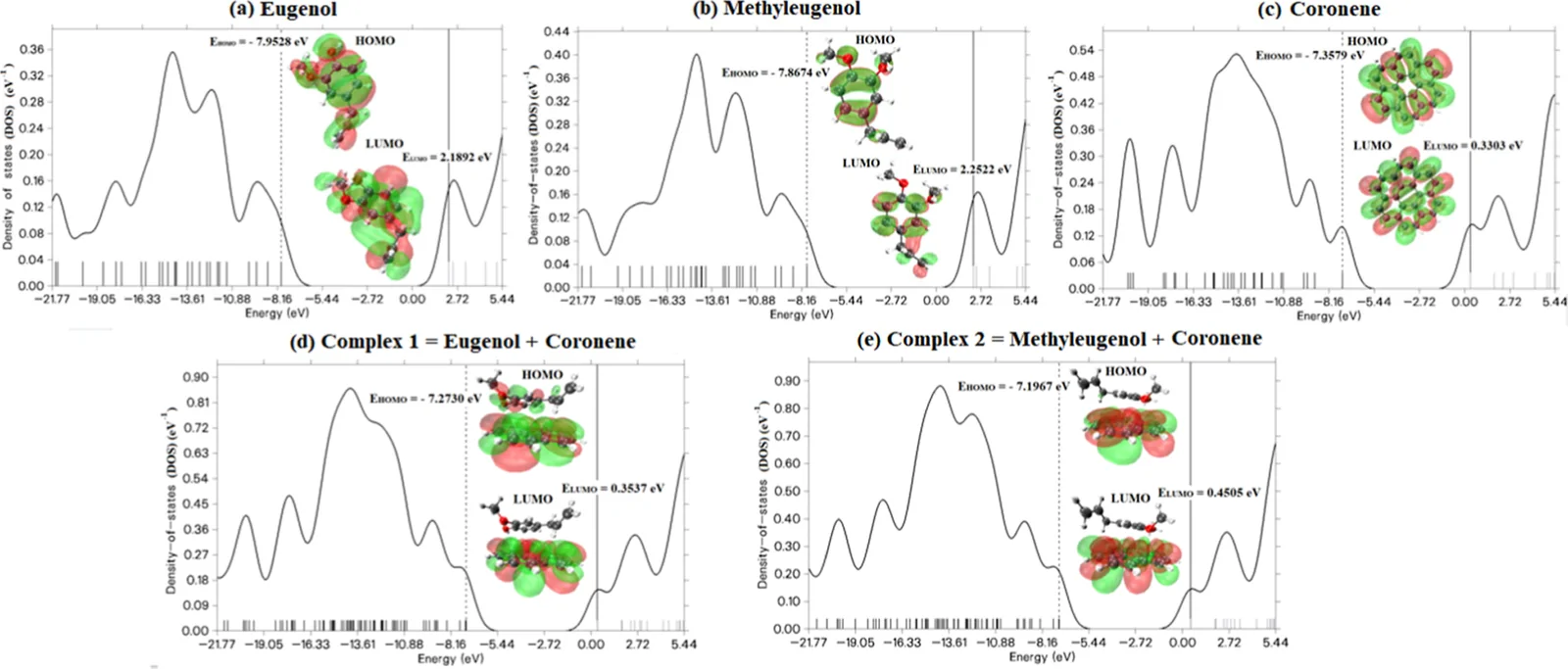
OMFs (insets) and DOS for (a) eugenol, (b) methyleugenol, (c) coronene, (d) complex 1 = eugenol + coronene, and (e) complex 2 = methyleugenol + coronene.

Figure 3(a,b) show that the HOMO (−7.8674 eV and −7.9528 eV) of both molecules is mainly distributed over the aromatic ring and oxygen-containing functional groups, indicating that these regions are the primary electron-donating sites. In contrast, the LUMO (2.1892 and 2.522 eV) is predominantly localized on the aromatic ring and allyl substituent (H_2_C=CH–CH_2_), suggesting their involvement in electron-accepting processes. A comparison between the two molecules reveals that methyleugenol exhibits a slightly higher HOMO energy (−7.8674 eV) and a smaller HOMO–LUMO energy gap (10.120 eV) than eugenol (−7.9528 and 10.142 eV). This behavior can be attributed to the presence of an additional methoxy group (−OCH_3_), which increases the electron density and polarizability of the molecule. Consequently, methyleugenol is expected to possess a greater electron-donating capability and enhanced electronic interaction with the coronene surface. The spatial overlap between the frontier orbitals and the π-conjugated system of the nanoribbon favors charge transfer and π–π interactions, which are consistent with the adsorption energies and charge-transfer results obtained for the adsorption complexes.

In Figure 3c, the FMOs (HOMO and LUMO) and DOS of the coronene were analyzed to investigate its electronic structure and potential interaction with adsorbed molecules. The HOMO (−7.3579 eV) is mainly delocalized along the π-conjugated carbon framework of the nanographene (graphene nanoflakes), reflecting the extended electronic conjugation characteristic of graphene-based materials. Similarly, the LUMO (0.3303 eV) is distributed over the carbon backbone, indicating that both occupied and unoccupied electronic states are readily available for interactions with adsorbate molecules. The positive Kohn–Sham LUMO energies are commonly observed in finite DFT calculations and should not be interpreted as physically occupied excited states. Instead, they reflect the approximate nature of Kohn–Sham orbital energies and do not imply spontaneous electron emission into the vacuum.

The delocalized nature of the FMOs observed in coronene is a hallmark of graphene-based π-conjugated systems, such as 0-D nanographene (graphene nanoflakes), which can be regarded as 2-D extensions of PAHs. It establishes a direct structural and electronic bridge to 1-D armchair graphene nanoribbons (AGNRs), which are commonly synthesized from polyphenylene precursors through lateral fusion.^54^

This relationship is defined by several key physical principles: ***(1) Quantum Confinement and Edge Structure:*** *(a) Cut-outs from Graphene:* Both coronene and AGNRs represent finite-sized segments of a graphene sheet. *(b) Armchair Symmetry:* Coronene’s perimeter can be viewed as an intersection of armchair-terminated edges. Because AGNRs are cut along this specific symmetry, the orbital phase distributions and nodal patterns smoothly map from the molecular disk to the extended nanoribbon.^55^ ***(2)*** π ***-Conjugation and Delocalization:*** *(a) Continuous* π*-System:* In coronene (C_24_H_12_), the overlapping p_*z*_ orbitals create a cyclic fully conjugated π-network. *(b) Translational Extension:* When transitioning to AGNRs, the expansion of the π-system along one axis preserves this aromatic delocalization but transforms the discrete molecular energy levels into continuous 1-D energy bands.^56^ ***(3) Optoelectronic Behavior:*** *(a) Size-Dependent Band Gap:* In both systems, the HOMO–LUMO gap scales inversely with size or ribbon width. Coronene effectively models the quantum-confined band gap opening characteristic of narrow AGNRs. *(b) Transition to Bulk:* These structural similarities allow researchers to study how bulk graphene properties emerge out of isolated molecular units.^54^

The OMFs facilitate charge-transfer processes and promotes electronic coupling between the coronene surface and aromatic molecules such as eugenol and methyleugenol. In particular, the overlap between the π-orbitals of the adsorbates and those of the coronene favors π–π stacking interactions, which contribute to the stability of the adsorption complexes. Furthermore, the relatively small HOMO–LUMO gap of coronene (7.688 eV), compared with those of eugenol (10.142 eV) and methyleugenol (10.120 eV), is expected to enhance its sensitivity to molecular adsorption, making it a promising candidate for sensing and molecular recognition applications.

The OMFs and DOS of the adsorption complexes, namely eugenol/coronene (complex 1) and methyleugenol/coronene (complex 2), were analyzed to investigate the electronic interactions between the adsorbate molecules and the coronene. As shown in Figure 3(d,e), the HOMO (−7.2730 eV and −7.1967 eV) and LUMO (0.3517 and 0.4505 eV) orbitals are predominantly distributed over the coronene framework, while a noticeable contribution from the adsorbed molecules is also observed, indicating electronic coupling between the π-conjugated systems of the adsorbates and the nanographene.

For both complexes, the HOMO is mainly localized on the aromatic regions of the adsorbate and the adjacent carbon atoms of the coronene surface, whereas the LUMO extends over the coronene and partially over the molecular structure. This orbital distribution suggests the occurrence of charge delocalization across the adsorbate–substrate interface, facilitating charge-transfer processes upon adsorption.

Furthermore, the adsorption of eugenol and methyleugenol slightly modifies the frontier orbital energies of the pristine coronene, resulting in changes in the HOMO–LUMO energy gap (7.625 eV for complex 1 and 7.647 eV for complex 2). Among the two complexes, eugenol/coronene is expected to exhibit stronger electronic interactions due to the higher polarizability and electron-donating ability of methyleugenol, in agreement with its slightly larger E_Ads_ and charge-transfer magnitude.

The HOMO and LUMO representations provide insights into the interaction of each molecule with graphene. In the case of methyleugenol, possibly due to its different adsorption orientations on the coronene surface, no significant contribution to the FMOs is observed, suggesting a predominantly physisorption-driven interaction. In contrast, for the eugenol–coronene complex, the slight contribution of the eugenol molecule to the HOMO indicates that it retains part of its electron-donating character, which may be associated with a stronger interaction with the coronene surface.

Despite the greater contribution of eugenol to the FMOs compared with methyleugenol, both molecules exhibit only a minor influence on the HOMO and LUMO distributions, suggesting that the adsorption is predominantly governed by physisorption, with π–−π interactions and van der Waals forces playing the dominant role.

Overall, the HOMO and LUMO distributions reveal effective electronic communication between the adsorbate molecules and the coronene surface, supporting the *E*_Ads_ and charge-transfer results and confirming the potential of coronene-based nanostructures for molecular recognition and sensing applications.

### 3.5 Molecular Electrostatic Potential (MEP) Map

The MEP maps of eugenol, methyleugenol, coronene, and their corresponding adsorption complexes were analyzed to identify the charge distribution and the most reactive regions involved in the adsorption process, see Figure 4a–e. The MEP surfaces provide valuable information about electrophilic and nucleophilic sites, where regions of negative electrostatic potential (NEP–shown in red) correspond to electron-rich areas, while regions of positive electrostatic potential (PEP–shown in blue) indicate electron-deficient areas. The electrostatic potential values range from −5.000 × 10^–2^ to +5.000 × 10^–2^ a.u.

**Figure 4:**
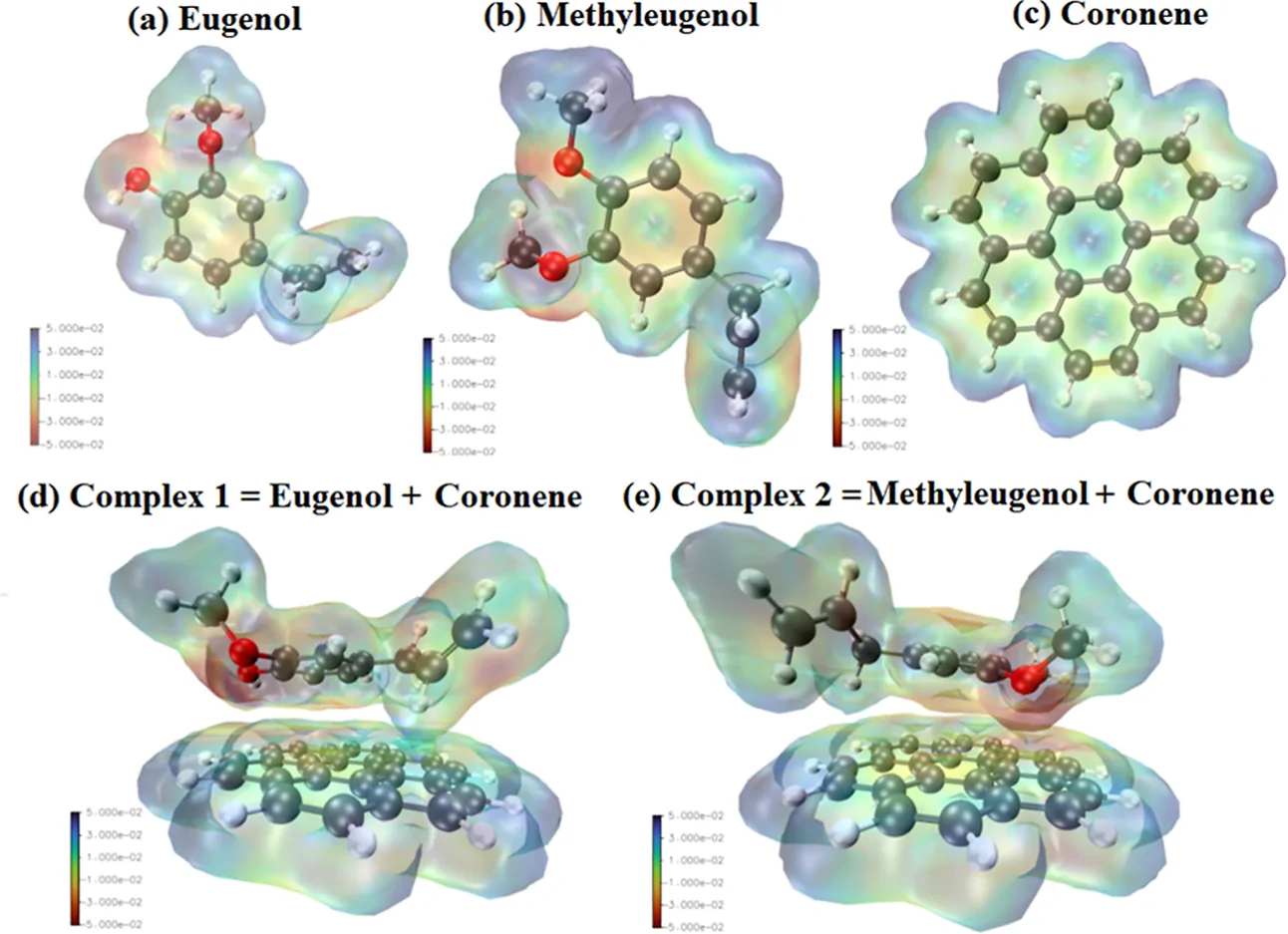
Molecular electrostatic potential (MEP) maps of (a) eugenol, (b) methyleugenol, (c) coronene, (d) eugenol/coronene (complex 1), and (e) methyleugenol/coronene (complex 2). Red and blue regions correspond to electron-rich (negative potential) and electron-deficient (positive potential) areas, respectively.

Figure 4a shows that for eugenol, the most NEP is mainly localized around the oxygen (O) atoms of the hydroxyl (OH) and methoxy (−OCH_3_) groups, reflecting their high electron density and strong nucleophilic character. Similarly, in Figure 4b, methyleugenol exhibits pronounced NEP around its two methoxy groups (−OCH_3_), with a more homogeneous electron distribution over the aromatic ring due to the additional electron-donating substituent. As a result, methyl eugenol generally presents a higher electronic polarizability than eugenol.

In Figure 4c, the MEP map of the coronene shows a relatively uniform electrostatic potential distribution over the π-conjugated carbon framework, indicating electronic delocalization throughout the nanographene. Slight variations in potential may be observed near the hydrogen-terminated edges due to local polarization effects.

Upon adsorption, the MEP maps of the eugenol/coronene (complex 1) and methyleugenol/coronene (complex 2) complexes reveal a redistribution of electron density at the adsorbate–substrate interface, see Figure 4(d,e). The electron-rich regions associated with the oxygen-containing functional groups interact with the delocalized π-electron system of coronene, leading to local changes in the electrostatic potential. These modifications are consistent with the charge-transfer analysis and E_Ads_ results, confirming the occurrence of weak but effective electronic interactions between the molecules and the nanographene surface.

As shown by the charge distribution maps, no significant electronic interaction is observed between the adsorbate and the coronene surface. Only minor localized changes in the charge distribution are detected, indicating weak intermolecular interactions, such as van der Waals and electrostatic interactions. This behavior is consistent with a physisorption mechanism.

Overall, the MEP analysis indicates that the oxygen-containing functional groups of eugenol and methyleugenol play a key role in the adsorption process, while the extended π-conjugated structure of coronene promotes charge delocalization and stabilizes the adsorption complexes.

### 3.6 Global Reactivity Parameters (GRPs)

The GRPs were calculated from the FMOs energies (E_HOMO_ and E_LUMO_) using Koopmans’ approximation to evaluate the chemical reactivity and electronic stability of eugenol, methyleugenol, coronene, and their corresponding adsorption complexes, see Table 6. According to Koopmans’ theorem, the *IP* and *EA* are approximated by the negative values of the *E*_*HOMO*_ and *E*_*LUMO*_, respectively. The remaining descriptors are obtained from the following relationships given by eqs 2–9).

**Table 6:** Calculated GRPs for Neutral Systems: Eugenol, Methyleugenol, Coronene, and the Corresponding Adsorption Complexes

systems/GRPs	eugenol (eV)	methyleugenol (eV)	coronene (eV)	complex 1 = eugenol + coronene (eV)	complex 2 = methyleugenol + coronene (eV)
*E*_*HOMO*_	–7.9528	–7.8647	–7.3579	–7.2730	–7.1967
*E*_*LUMO*_	2.1892	2.2522	0.3303	0.3537	0.4505
*E*_*gap*_	10.1420	10.1169	7.6882	7.6267	7.6472
*IP*	7.9528	7.8647	7.3579	7.2730	7.1967
*EA*	–2.1892	–2.2522	–0.3303	–0.3537	–0.4505
μ	–2.8818	–2.8062	–3.5138	–3.4596	–3.3731
χ	2.8818	2.8062	3.5138	3.4596	3.3731
η	5.0710	5.0584	3.8441	3.8133	3.8236
*S*	0.1025 eV^–1^	0.1028 eV^–1^	0.1352 eV^–1^	0.1363 eV^–1^	0.1359 eV^–1^
ω	0.8512	0.8095	1.6692	1.6313	1.5462
Δ*N*_*max*_	0.5683	0.5547	0.9141	0.9140	0.8821

Table 6 shows that the HOMO–LUMO gap (*E*_*gap*_) is higher for the isolated molecules (10.142 and 10.1169 eV), consistent with more electronically stable and less reactive systems, whereas lower values are obtained for coronene (7.6882 eV) and the complexes (7.6267 and 7.6472 eV), suggesting enhanced chemical reactivity and polarizability, which are favorable for adsorption and sensing applications.

*IP* and *EA* are higher (7.9528 and 7.8647 eV) and more negative (−2.1892 and −2.2522 eV) for the isolated molecules, indicating greater stability against electron loss and a lower tendency to accept electrons. In contrast, lower *IP* and less negative *EA* values are observed for coronene (7.3579 eV and −0.3303 eV) and the adsorption complexes (7.2730 and 7.1967 eV and −0.3537 and −0.4505 eV), suggesting easier ionization, enhanced chemical reactivity, and a comparatively increased ability to accept electrons during interaction processes.

The μ is less negative for the isolated molecules (−2.8818 and −2.8062 eV), indicating a greater tendency to donate electrons, whereas more negative values are found for coronene (−3.5138 eV) and the complexes (−3.4596 and −3.3732 eV), reflecting higher electronic stability and reduced tendency for spontaneous electron loss.

Similarly, the χ and ω are lower for the isolated molecules (2.8818 and 2.8062 eV and 0.8512 and 0.8095 eV), indicating a weaker tendency to attract electrons and lower electrophilic character. In contrast, higher χ and ω values are obtained for coronene (3.5138 and 1.6692 eV) and the adsorption complexes (3.4596 and 3.3731 eV and 1.6313 and 1.5462 eV), suggesting a stronger electrophilic character, greater tendency to attract electron density, and enhanced ability to accept electrons during chemical interactions.

The isolated molecules exhibit lower values for *S* (0.1025 and 0.1028 eV^–1^) and Δ*N*_*max*_ (0.5683 and 0.5547), indicating reduced polarizability and reactivity and limited electron-transfer ability, whereas higher values for *S* and Δ*N*_*max*_ are obtained for coronene (0.1352 eV^–1^ and 0.9141) and the complexes (0.1363 eV^–1^ and 01359 eV^–1^, 0.9140 and 0.8821), reflecting increased electronic softness, enhanced charge redistribution capability and participation in charge-transfer interactions during adsorption.

The calculated GRPs provide valuable information regarding the electronic behavior of the investigated systems. In general, molecules with higher HOMO energies exhibit a greater tendency to donate electrons, whereas lower LUMO energies indicate an enhanced ability to accept electrons. Likewise, a smaller HOMO–LUMO energy gap and lower chemical hardness (η) are associated with higher chemical reactivity and greater electronic polarizability.

The adsorption of eugenol and methyl eugenol on the coronene surface slightly modifies these descriptors, indicating electronic interactions between the adsorbate molecules and the nanographene. These variations are consistent with the *E*_Ads_, charge-transfer analysis, FMO distributions, and MEP maps, confirming the formation of stable adsorption complexes through predominantly physisorption interactions.

### 3.7 IR/Raman Vibrational Spectrum

In addition to the structural relaxation analysis, the dynamical stability of the isolated molecules, coronene, and the adsorption complexes was evaluated through DFT-calculated IR and Raman vibrational spectra, as shown in Figure 5(a–e) and Figure 6(a–e). The discussion focus on the assignment of the vibrational modes, the adsorption-induced changes in the vibrational spectra, and the confirmation of the structural stability of the proposed systems.

**Figure 5:**
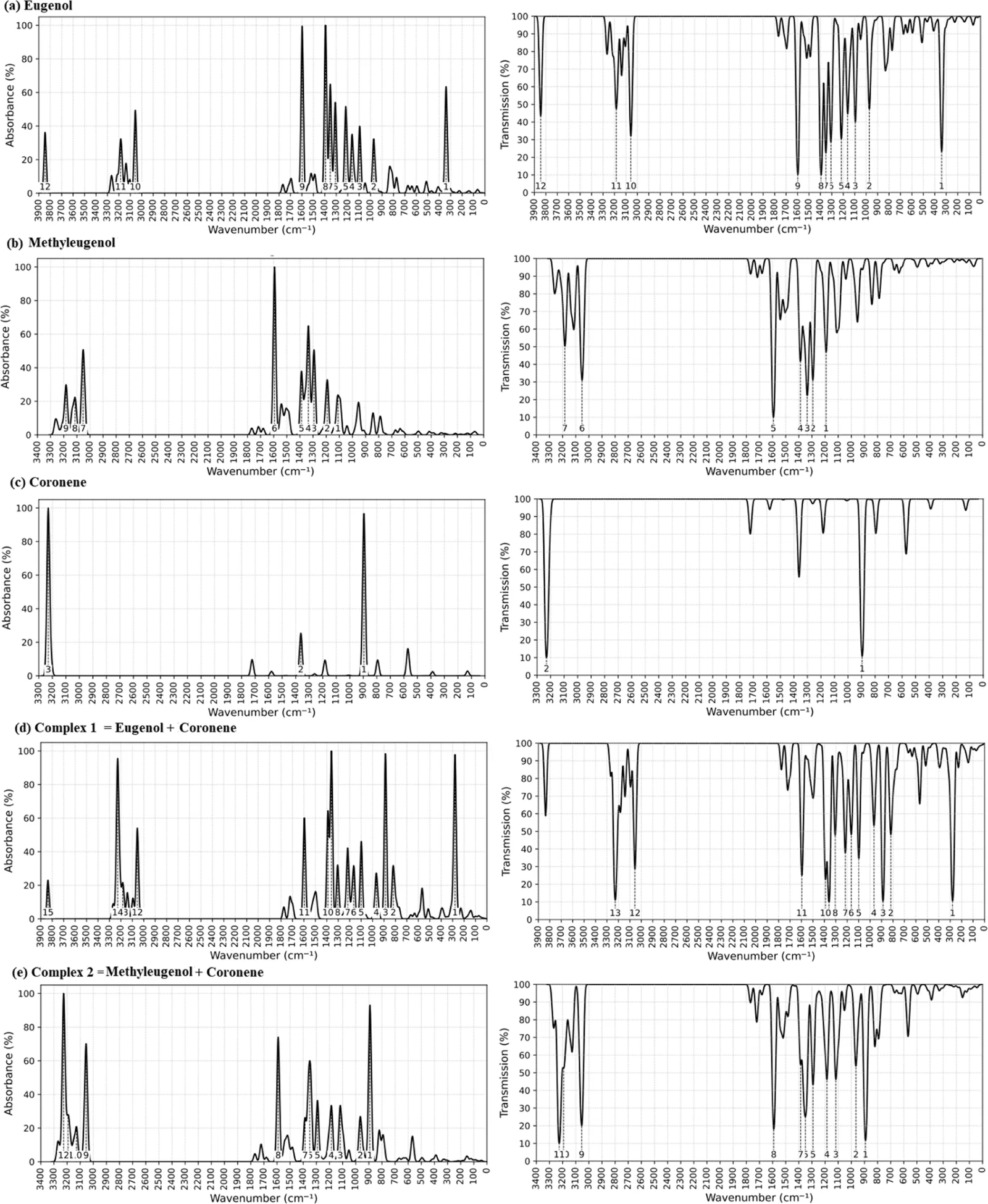
IR absorbance and transmittance spectra for (a) eugenol, (b) methyleugenol, (c) coronene, (d) complex 1 = eugenol + coronene, and (e) complex 2 = methyleugenol + coronene.

**Figure 6:**
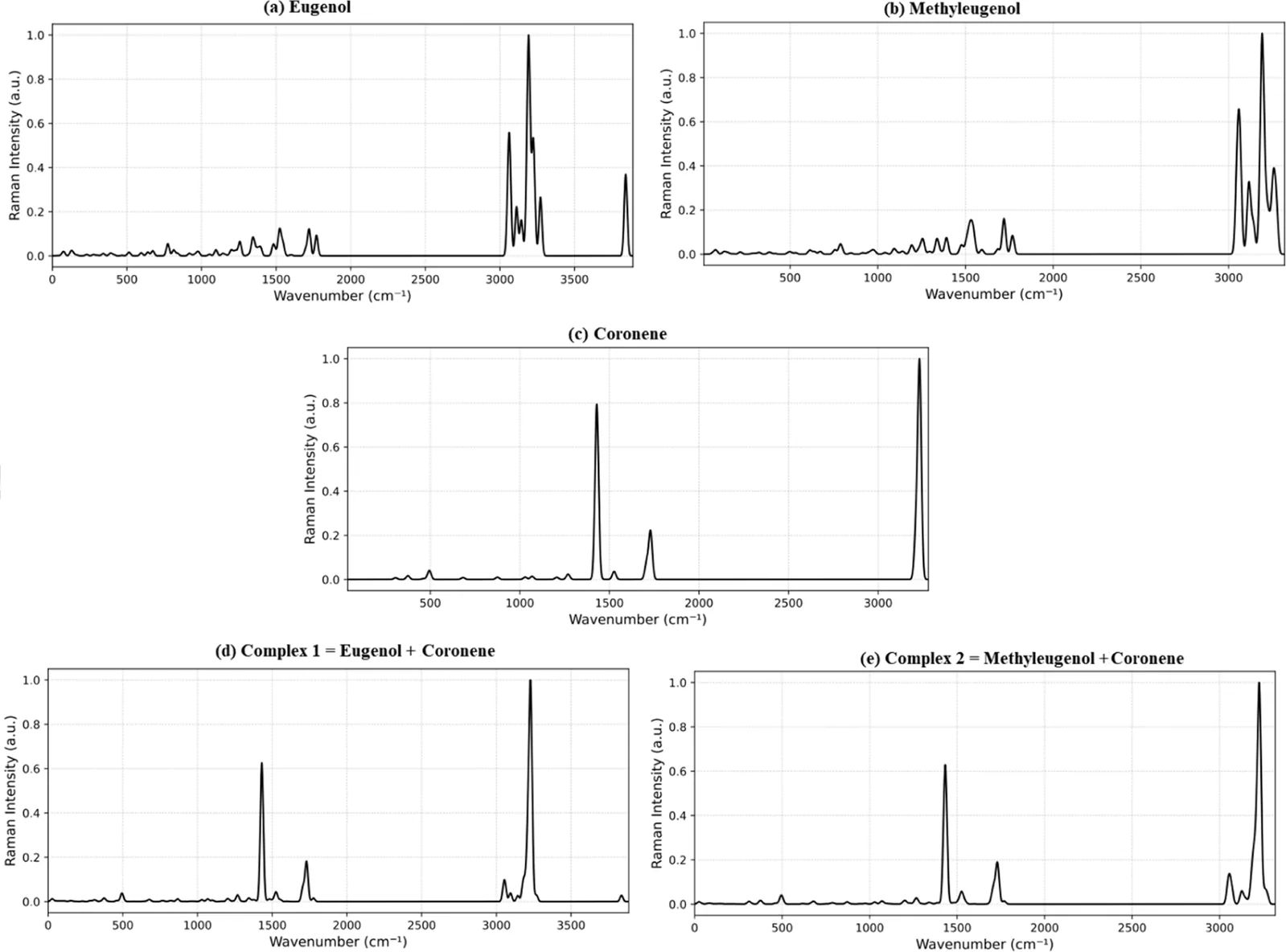
Raman spectrum for (a) eugenol, (b) methyleugenol, (c) coronene, (d) complex 1 = eugenol + coronene, and (e) complex 2 = methyleugenol + coronene.

All computed vibrational frequencies are real with no imaginary modes detected, confirming that the optimized geometries correspond to true local minima on the potential energy surface. This result demonstrates the dynamic stability of the proposed structures within the harmonic approximation at 0 K, where thermal effects are neglected and the systems are described in their ground-state equilibrium configurations.

The high-intensity vibrational bands observed in the IR and Raman spectra are associated with strongly dipole- and polarizability-active vibrational modes, reflecting the robustness of chemical bonding within the optimized structures. Although IR and Raman spectra do not replace phonon dispersion calculations for extended periodic systems, they provide reliable evidence of local dynamic stability for the finite graphene nanoribbon models investigated in this work. In contrast, phonon dispersion calculations are generally employed to assess the dynamic stability of infinite periodic materials, where imaginary phonon modes indicate structural instabilities or soft vibrational modes. Likewise, finite-temperature stability is commonly evaluated by *ab initio* molecular dynamics (AIMD) simulations, typically performed at 300 K or higher, to verify the structural integrity of the systems under realistic thermal conditions.

Therefore, the absence of imaginary vibrational frequencies, together with favorable energies, provides consistent evidence of the energetic, structural, and local dynamical stability of the proposed adsorption systems. Furthermore, the calculated IR and Raman spectra are highly sensitive to structural features such as the bonding environment, edge configuration, ribbon width, and adsorption-induced perturbations, making them valuable tools for characterizing graphene-based nanostructures and their adsorption complexes.

Figure 5(a–e) characterizes the vibrational signatures of the systems obtained from IR spectroscopy represented by absorbance and transmittance. The difference between the absorbance and transmittance spectra lies only in the representation of the data; the vibrational frequencies remain identical in both spectra. The absorption bands appear as upward peaks. The higher the peak intensity, the greater the IR absorption at that wavenumber. The transmission bands appear as downward peaks (decreases in the transmittance). The lower is the transmittance, the greater is the IR absorption.

Mathematically, absorbance (*A*) is related to transmittance (*T*) by eq 11

12 $$A=-\log_{10}\left(T\right)$$

where *T* = *I/I*_*0*_ is the transmittance, *I* is the transmitted intensity, and *I*_*0*_ is the incident intensity.

Figure 5a shows that the IR spectrum of eugenol is dominated by the characteristic vibrational modes of its functional groups. The broad band observed between 3800 and 3900 cm^–1^ is assigned to the O–H stretching vibration of the hydroxyl group, whereas the aromatic and aliphatic C–H stretching modes appear in the 3000–3300 cm^–1^ region. Vibrations associated with the aromatic ring, particularly the C=C stretching modes, are observed between 1500 and 1800 cm^–1^, while the C–O stretching vibrations of the phenolic group occur in the 1100–1400 cm^–1^ range.

Methyleugenol exhibits a vibrational profile similar to that of eugenol; however, the substitution of the hydroxyl group by a methoxy group eliminates the characteristic O–H stretching band observed in the IR spectrum of eugenol, see Figure 5b. Instead, vibrational modes associated with the O–CH_3_ group, particularly the C–O stretching vibrations and CH_3_ deformation modes, become more prominent. The aromatic and aliphatic C–H stretching modes appear in the 3000–3300 cm^–1^ region. Vibrations associated with the aromatic ring, particularly the C=C stretching modes, are observed between 1500 and 1800 cm^–1^, while the C–O stretching vibrations of the phenolic group occur in the 1100–1400 cm^–1^ range.

The vibrational spectrum of the coronene is primarily characterized by vibrational modes associated with the sp^2^-hybridized carbon framework that induce a change in the molecular dipole moment are infrared-active. Since coronene possesses a high degree of symmetry (point group D_6h_), many vibrational modes are IR-inactive, resulting in a relatively small number of infrared absorption bands, see Figure 5c. In the IR spectrum, bands corresponding to the C=C and C–C stretching vibrations of the graphitic lattice are observed in the 1300–1400 cm^–1^ and 1700–1800 cm^–1^ ranges, together with C–H stretching, in-plane and out-of-plane C–H bending modes arising from the hydrogen atoms that passivate the nanoribbon edges in the 3200–3300 cm^–1^, 1300–1400 cm^–1^ and 850–950 cm^–1^ ranges, respectively.

Figure 5d shows the adsorption configuration of eugenol on the coronene surface, the main vibrational modes of both species remain essentially preserved, indicating that the chemical structures of the adsorbate and the substrate are not significantly altered during the adsorption process. Nevertheless, slight shifts in the positions and relative intensities of several vibrational bands, particularly those associated with the O–H and C–O functional groups and the aromatic C=C stretching modes, provide evidence of an interaction between eugenol and the coronene surface. These spectral changes are consistent with localized charge redistribution and weak intermolecular interactions between the adsorbate and the adsorbent, supporting a predominantly physisorption-driven adsorption mechanism.

The methyleugenol–coronene complex exhibits a similar vibrational behavior, preserving nearly all the characteristic vibrational features of the isolated components, see Figure 5e. The changes observed in the vibrational frequencies and band intensities are even less pronounced than those found for the eugenol–coronene complex, indicating a weaker interaction with the coronene surface. This behavior is consistent with the smaller charge redistribution, the negligible contribution to the FMOs, and the predominantly physisorption-driven nature of the adsorption process revealed by the electronic structure analyses.

Figures S3 and S4 presents the IR spectra of the three local-minimum adsorption geometries (extra 1, extra 2, and extra 3) for complexes 1 (eugenol + coronene) and 2 (methyleugenol + coronene), respectively. The optimized geometries were confirmed to be true minima by the absence of imaginary frequencies (i.e., negative wavenumbers).

Figure 6a-e characterizes the vibrational signatures of the systems obtained from Raman spectroscopy. Figure 6a shows that the Raman spectrum is dominated by vibrational modes associated with the aromatic ring owing to the high polarizability of the π-conjugated system. The bands assigned to the C = C stretching vibrations of both the aromatic ring and the allyl side chain are particularly intense, reflecting the extended electronic conjugation within the molecule.

Similarly, the Raman spectrum for methyl eugenol is dominated by vibrational modes of the aromatic ring and the allyl side chain, as shown in Figure 6b. However, slight variations in the relative intensities of several Raman bands are observed compared to eugenol, reflecting the redistribution of electron density caused by the methoxy substituent while preserving the overall vibrational profile of the molecule.

In Figure 6c exhibits the Raman spectrum of coronene that is characterized by the vibrational modes of the graphitic carbon framework. Vibrations associated with the sp^2^-hybridized carbon network exhibit high intensities, reflecting the strong polarizability of the delocalized π-electron system. Depending on the nanoribbon geometry, spectral features analogous to the characteristic graphite (G) and defect (D) bands of graphitic materials may also be identified, with their positions and intensities influenced by the ribbon width and hydrogen-passivated edges. The bands for C–C and C=C stretching vibrations of the graphitic lattice are observed in ≈1450 cm^–1^ and ≈1550 cm^–1^, which C=C band is analogous to the G band of graphene, together with C–H stretching modes arising from the hydrogen atoms that passivate the nanoribbon edges in ≈3500 cm^–1^ and ring deformation/C–C–C bending vibrations in ≈500 cm^–1^.

In Figure 6d slight variations in the intensities of Raman bands associated with the π-conjugated system indicate weak electronic coupling between eugenol and the coronene surface. The preservation of the characteristic vibrational modes, together with the absence of new Raman-active bands, suggests that adsorption occurs without the formation of new covalent bonds.

In the methyleugenol–coronene complex, the characteristic Raman modes of coronene remain essentially unchanged, while the vibrational bands of methyleugenol exhibit only minor perturbations, see Figure 6e. These results further support the absence of significant electronic hybridization and confirm that the interaction is predominantly governed by weak noncovalent forces.

The IR and Raman spectra of all systems exhibit only real vibrational frequencies, with no imaginary modes, confirming that the optimized geometries correspond to local minima on the potential energy surface. The preservation of the characteristic vibrational modes of the molecules after adsorption, accompanied only by slight frequency shifts and changes in band intensities, indicates that the adsorption process is predominantly governed by physisorption through π–π interactions, van der Waals forces, and weak electrostatic interactions, without the formation or breaking of covalent bonds. This behavior is consistent with the calculated adsorption energies, the limited charge transfer, and the minor perturbation of the frontier molecular orbitals observed for the adsorption complexes.

## 4 Conclusions

In this work, a comparative first-principles investigation of the adsorption of eugenol and methyleugenol on coronene was performed using DFT, with coronene employed as a simplified finite molecular model of a local graphene-like π-conjugated surface. Both phenylpropanoids preferentially adopted parallel configurations relative to the coronene plane, maximizing π–π interactions between their aromatic rings and the delocalized π-electron system of the carbon surface. The calculated equilibrium adsorption distances and negative adsorption energies (E_Ads_) indicate energetically favorable complex formation within the electronic-structure framework adopted within the physisorption regime; however, they should not be taken as evidence of thermodynamic favorability because thermal, entropic, zero-point-energy, and other contributions to Gibbs free energy were not evaluated.

The uncorrected E_Ads_ values were −0.645 eV for eugenol/coronene and −0.672 eV for methyleugenol/coronene, indicating moderate physisorption and a slightly stronger interaction for methyleugenol. Benchmark calculations demonstrated a significant dependence of E_Ads_ on the basis set, with the relative BSSE contribution decreasing from approximately 29–33% with 6–31G­(d) to approximately 4.8–5.2% with def2-TZVPD basis set. The BSSE-corrected E_Ads_ obtained with the larger basis set ranged from −0.536 to −0.593 eV and are therefore considered the preferred quantitative values in the present study. Despite these quantitative differences, the relative adsorption behavior and the principal conclusions regarding the interaction mechanism remained unchanged.

Energy decomposition analysis (EDA) confirmed that dispersion is the dominant stabilizing contribution, followed by electrostatic and orbital interactions, supporting a predominantly noncovalent adsorption mechanism. The electronic analyses consistently indicated weak but measurable interactions between the phenylpropanoids and coronene, with only limited charge redistribution and modest changes in frontier molecular orbitals, DOS, and global reactivity descriptors. Vibrational analyses showed no imaginary frequencies and only minor changes in the characteristic IR and Raman features, further supporting the predominantly noncovalent nature of the complexes.

The comparison between eugenol and methyl eugenol indicates that methylation of the phenolic hydroxyl group produces a small computational trend toward somewhat stronger adsorption within the coronene model, without changing the fundamental physisorption mechanism. The additional comparison with a nonhydrogenated C_24_ graphene-like cluster yielded similar BSSE-corrected adsorption energies, supporting the consistency of the principal adsorption picture while also demonstrating that quantitative differences can arise from edge termination and finite-size effects.

Overall, coronene provides a useful and computationally tractable finite model for investigating local interactions between natural aromatic molecules and graphene-like π-conjugated carbon surfaces. Nevertheless, its finite size, hydrogen-terminated edges, and molecular nature of coronene must be considered when relating the results to extended graphene.

The present results provide molecular-level insights into the interaction of eugenol and methyleugenol with a finite graphene-like coronene model, which may be relevant to future studies of carbon-based sensing and corrosion inhibition systems. However, the present isolated molecular model does not directly represent the realistic sensor or corrosion environments. Further theoretical and experimental studies considering extended surfaces, substrates, solvents, competing adsorbates, temperature, and electrochemical conditions are therefore required to assess the practical sensing and corrosion inhibition performance of these molecules.

## Supplementary Material



## Data Availability

Data will be made available on request.

## Notes

Three local-minimum adsorption geometries (extra 1, extra 2, and extra 3) and their corresponding energies, energy decomposition analysis (EDA) method, energy decomposition analysis - natural orbital for chemical valence (EDA-NOCV) method, image of the most significant NOCV channel, and IR Spectra of the three local-minimum adsorption geometries (extra 1, extra 2, and extra 3) for Complexes 1 and 2. All energy components (E_h_) for Complexes 1 to 4 (PDF)
