# Supplementary material for: Theoretical Investigation of Adsorption of Eugenol and Methyleugenol on the Coronene Surface: A Simplified Finite Molecular Model of Graphene
Source: ACS Omega. 2026 Oct 9;11(41):63618–35. doi: 10.1021/acsomega.6c07744 (PMC13689255; doi:10.1021/acsomega.6c07744)
Supplement: Supplementary file 1 [file ao6c07744_si_001.pdf]

Supporting Information

Theoretical Investigation of Adsorption of Eugenol and Methyl-Eugenol on Coronene Surface: A Simplified Finite Molecular Model of Graphene

Yuri dos Santos Lima Pereira[a], Jonas Marinho Duarte[b], Alcy Favacho Ribeiro[a], Josiney Farias de Araújo[c], Alessandra Nacimento Braga[d], Marcos Vinícius da Silva Paula[e], Carlos Alberto Brito da Silva Jr.[d,*]

[a] *Faculdade de Química, UFPA, Ananindeua, 67113-901, Brazil*
[b] *Faculdade de Engenharia de Minas, UFOPA, Juruti, 68170-000, Brazil*
[c] *Pós-Graduação em Botânica Tropical, UFRA, Belém, 66077-530, Brazil*
[d] *Faculdade de Física, UFPA, Ananindeua, 67113-901, Brazil*
[e] *Faculdade de Engenharia de Materiais, UFPA, Ananindeua, 67113-901, Brazil*

*Corresponding Author: cabsjr@ufpa.br

Table S1 - The three local-minimum adsorption geometries (extra 1, extra 2, and extra 3) and the corresponding energies of Complex 1 = Eugenol + Coronene are presented below. The equilibrium distances between the eugenol molecule and coronene surface is in the range of 3.0–3.5 Å. Consider that 1 Hartree (E_h) = 27.2114 eV.

Complex 1	Geometry	Energy
extra 1	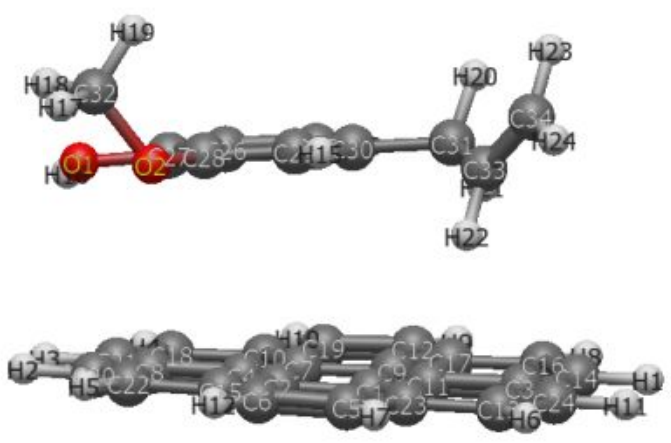	- 1,460.1727 Eh<br>- 39,733.3434 eV
extra 2	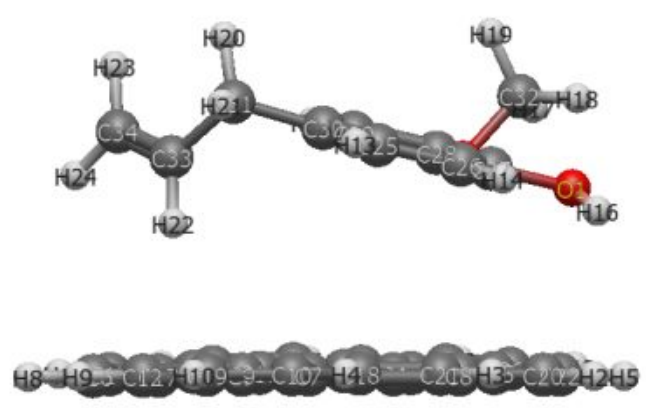	-1,460.1720 Eh<br>- 39,733.3243 eV
extra 3	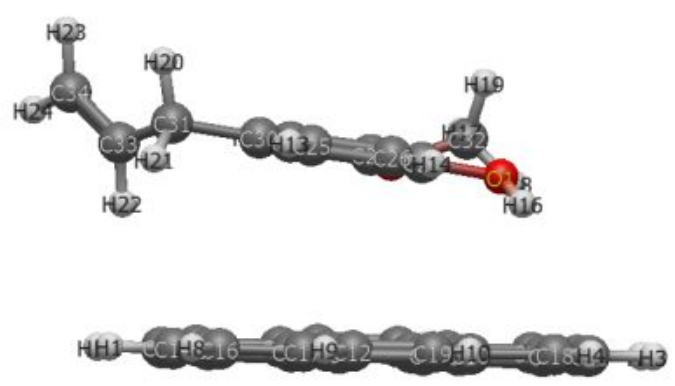	-1,460.1651 Eh<br>- 39,733.1366 eV

Table S2 - The three local-minimum adsorption geometries (extra 1, extra 2, and extra 3) and the corresponding energies of Complex 2 = Methyleugenol + Coronene are presented below. The equilibrium distances between the methyl-eugenol molecule and coronene surface is in the range of 3.0–3.6 Å. Consider that 1 Hartree (E_h) = 27.2114 eV.

Complex 2	Geometry	Energy
extra 1	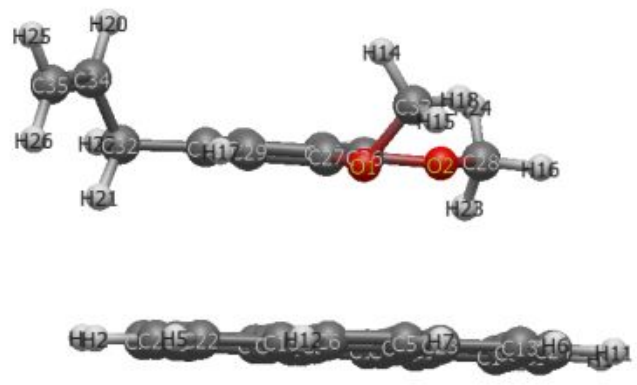	-1,499.4531 Eh<br>- 40,802.2180 eV
extra 2	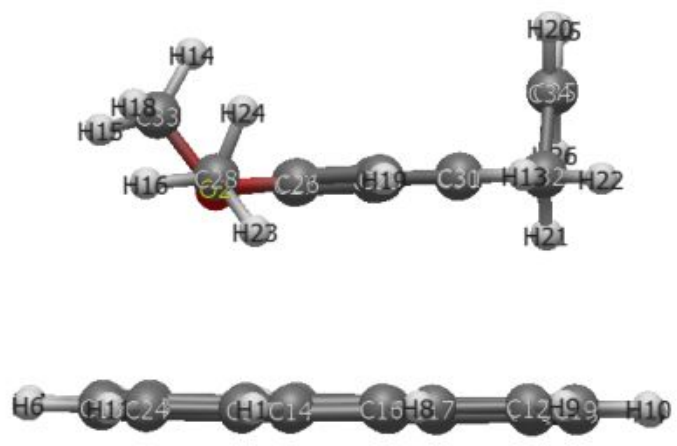	-1,499.4530 Eh<br>- 40,802.2153 eV
extra 3	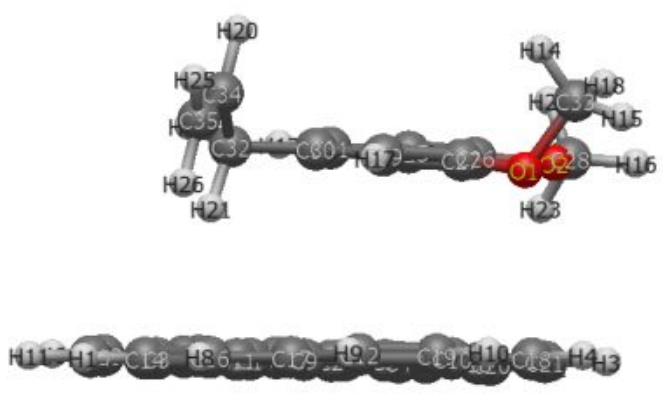	-1,499.4525 Eh<br>- 40,802.2017 eV

Table S3 - Energy decomposition analysis (EDA) Method of the Complex 1 (Eugenol + Coronene) bonds at DFT/wB97X-D3 6-31G(d). E_{Orb}, E_{Elet}, and E_{Disp} represent atractive forces.

**ENERGY DECOMPOSITION ANALYSIS (EDA) FOR COMPLEX 1 (EUGENOL + CORONENE)**<br>$\mathbf{E_{Bond} = E_{Orb} + E_{Elet} + E_{Disp} + E_{Pauli} + E^0_{XC}}$		
**Energy Term**	**Hartree (E_h)**	**Kcal/mol**
Bond Energy (E_{Bond})	-0.0183450838	-11.51
Orbital Energy (E_{Orb})	-0.0037880900	-2.38
Electrostatic Energy (E_{Elet})	-0.0097905044	-6.14
**Dispersion Energy (E_{Disp})**	**-0.0205517310**	**-12.90**
Pauli Energy (E_{Pauli})	0.0695298318	43.63
E^0_{XC}	-0.0537437667	-33.72

Table S4 - Energy decomposition analysis (EDA) Method of the Complex 2 (Methyleugenol + Coronene) bonds at DFT/wB97X-D3 6-31G(d).

**ENERGY DECOMPOSITION ANALYSIS (EDA) FOR COMPLEX 2 (METHYLEUGENOL + CORONENE)**<br>$\mathbf{E_{Bond} = E_{Orb} + E_{Elet} + E_{Disp} + E_{Pauli} + E^{0}_{XC}}$		
**Energy Term**	**Hartree**	**Kcal/mol**
Bond Energy (E_{Bond})	-0.0177677569	-11.15
Orbital Energy (E_{Orb})	-0.0040703608	-2.55
Electrostatic Energy (E_{Elet})	-0.0080576782	-5.06
**Dispersion Energy (E_{Disp})**	**-0.0225843278**	**-14.17**
Pauli Energy (E_{Pauli})	0.0745109474	46.76
ΔE^{0}_{XC}	-0.0575652668	-36.12

Table S5 - Energy decomposition analysis - Natural Orbital for Chemical Valence (EDA-NOCV) Method of the Complex 1 (Eugenol + Coronene) bonds at DFT/wB97X-D3 6-31G(d), where ΔE_k- total energy contribution ΔT_k- kinetic energy contribution, and ΔV_k- potential energy contribution.

**EDA-NOCV ANALYSIS**<br>$\boldsymbol{\Delta E_k = \Delta T_k + \Delta V_k}$				
**negative eigen. (e)**	**positive eigen. (e)**	$\boldsymbol{\Delta E_k}$ **(Kcal/mol)**	$\boldsymbol{\Delta T_k}$ **(Kcal/mol)**	$\boldsymbol{\Delta V_k}$ **(Kcal/mol)**
-0.0605974621	0.0605974621	-0.39	-10.77	10.38
-0.0503438097	0.0503438097	-0.29	3.30	3.01
-0.0465874443	0.0465874443	-0.33	-10.35	10.01
-0.0435396479	0.0435396479	-0.26	-5.58	5.32
-0.0322683059	0.0322683059	-0.17	-0.58	0.42

Figure S1 - Most significant NOCV channel image, with isovalue from 0.0001. Red regions represent electron density depletion, whereas blue regions represent electron density accumulation for Complex 1 (Eugenol + Coronene), where ΔE_k- total energy contribution, and ΔT_k- kinetic energy contribution, and ΔV_k- potential energy contribution.

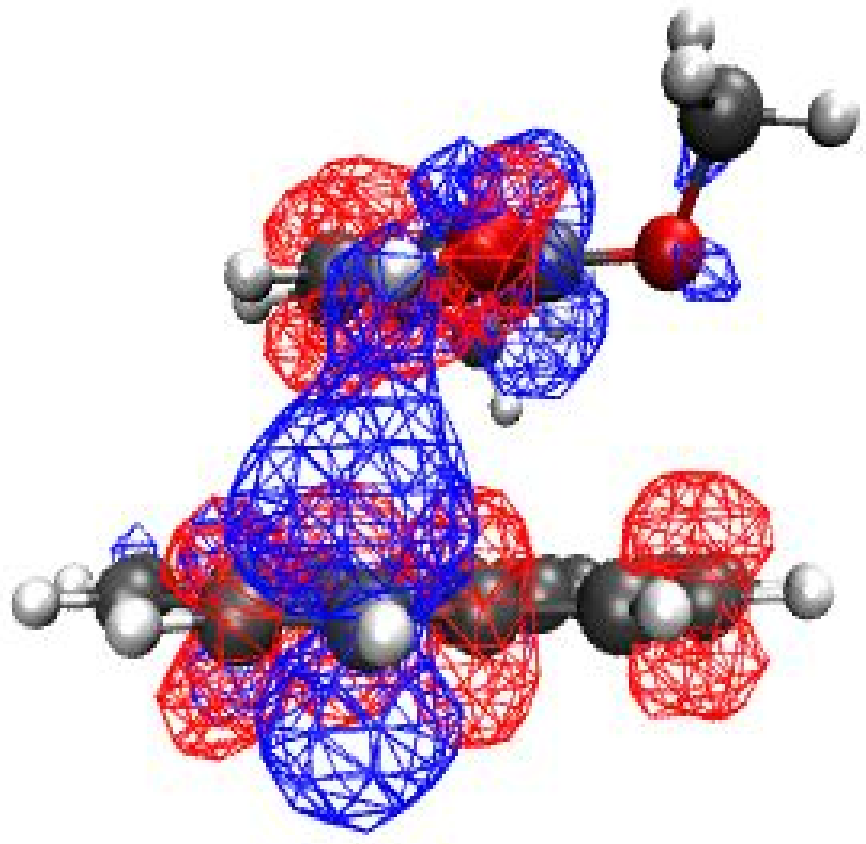

Table S6 - Energy decomposition analysis - Natural Orbital for Chemical Valence (EDA-NOCV) Method of the Complex 2 (Methyleugenol + Coronene) bonds at DFT/wB97X-D3 6-31G(d).

**EDA-NOCV ANALYSIS**				
**negative eigen. (e)**	**positive eigen. (e)**	**ΔE_k (Kcal/mol)**	**ΔT_k (Kcal/mol)**	**ΔV_k (Kcal/mol)**
-0.0565715504	0.0565715504	-0.39	-10.06	9.67
-0.0507178043	0.0507178043	-0.29	-8.51	8.22
-0.0424936620	0.0424936620	-0.32	-9.33	9.01
-0.0390483039	0.0390483039	-0.21	-7.23	7.01
-0.0359040146	0.0359040146	-0.19	-4.69	4.51

Figure S2 - Most significant NOCV channel image, with isovalue from 0.0001. Red regions represent electron density depletion, whereas blue regions represent electron density accumulation for Complex 2 (Methyleugenol + Coronene).

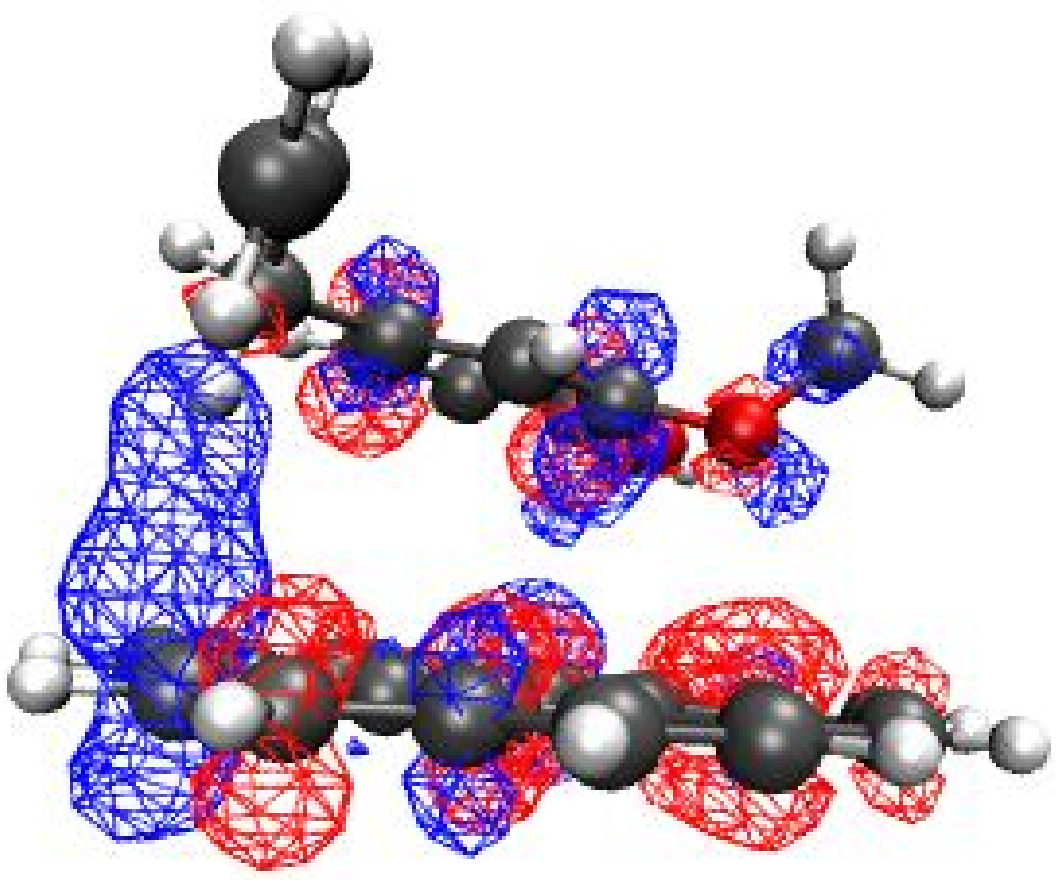

Figure S3 - IR Spectra of the three local-minimum adsorption geometries (extra 1, extra 2, and extra 3) for Complex 1 = Eugenol + Coronene are presented below. The optimized geometry was confirmed as true minima by the absence of imaginary frequencies (i.e., negative wavenumbers).

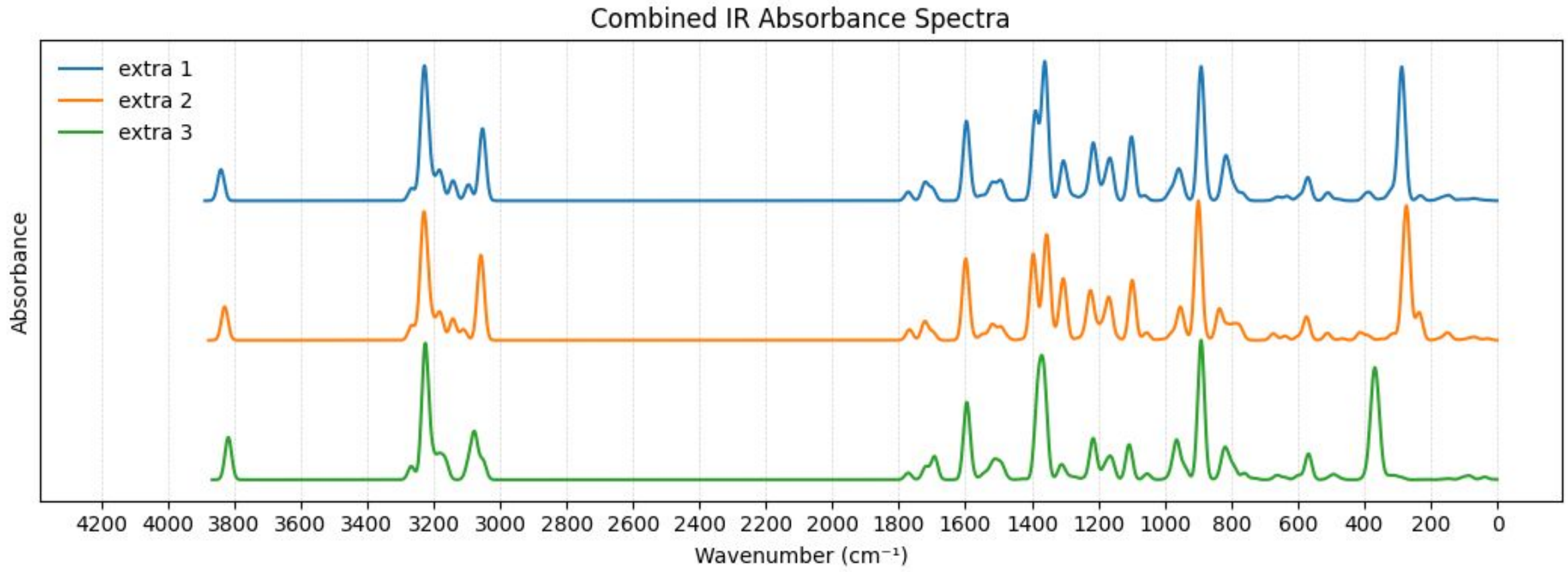

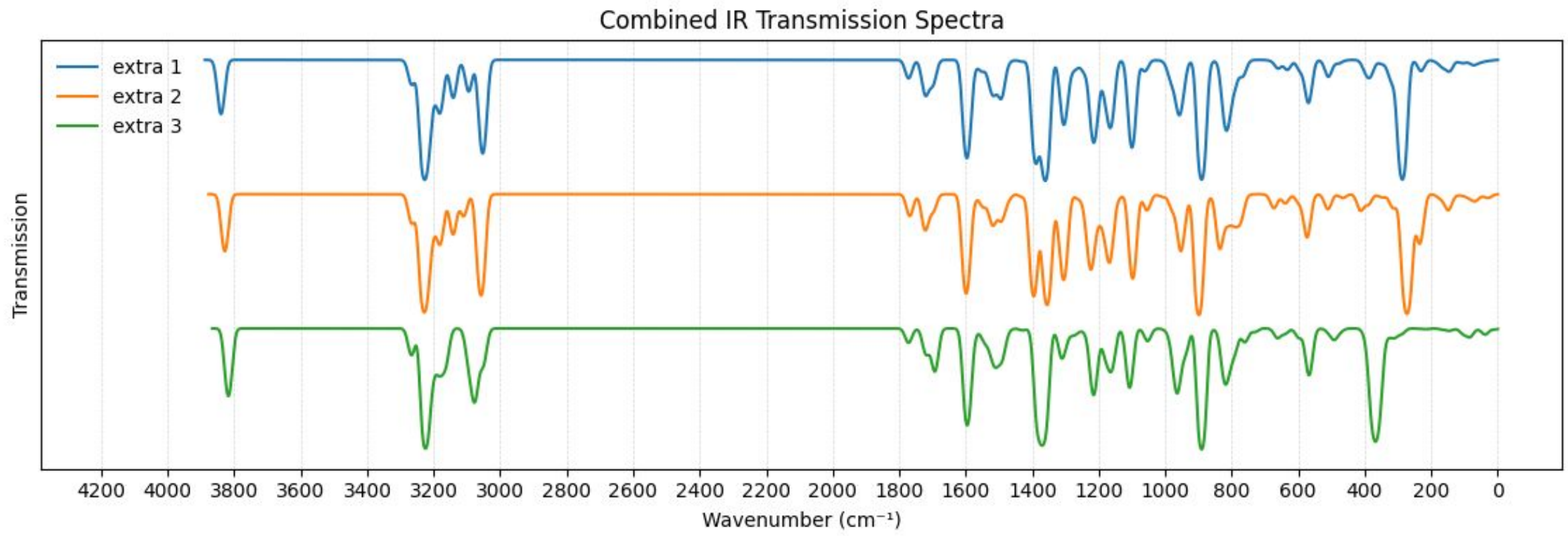

Figure S4 - IR Spectra of the three local-minimum adsorption geometries (extra 1, extra 2, and extra 3) for Complex 2 = Methyleugenol + Coronene are presented below. The optimized geometry was confirmed as true minima by the absence of imaginary frequencies (i.e., negative wavenumbers) .

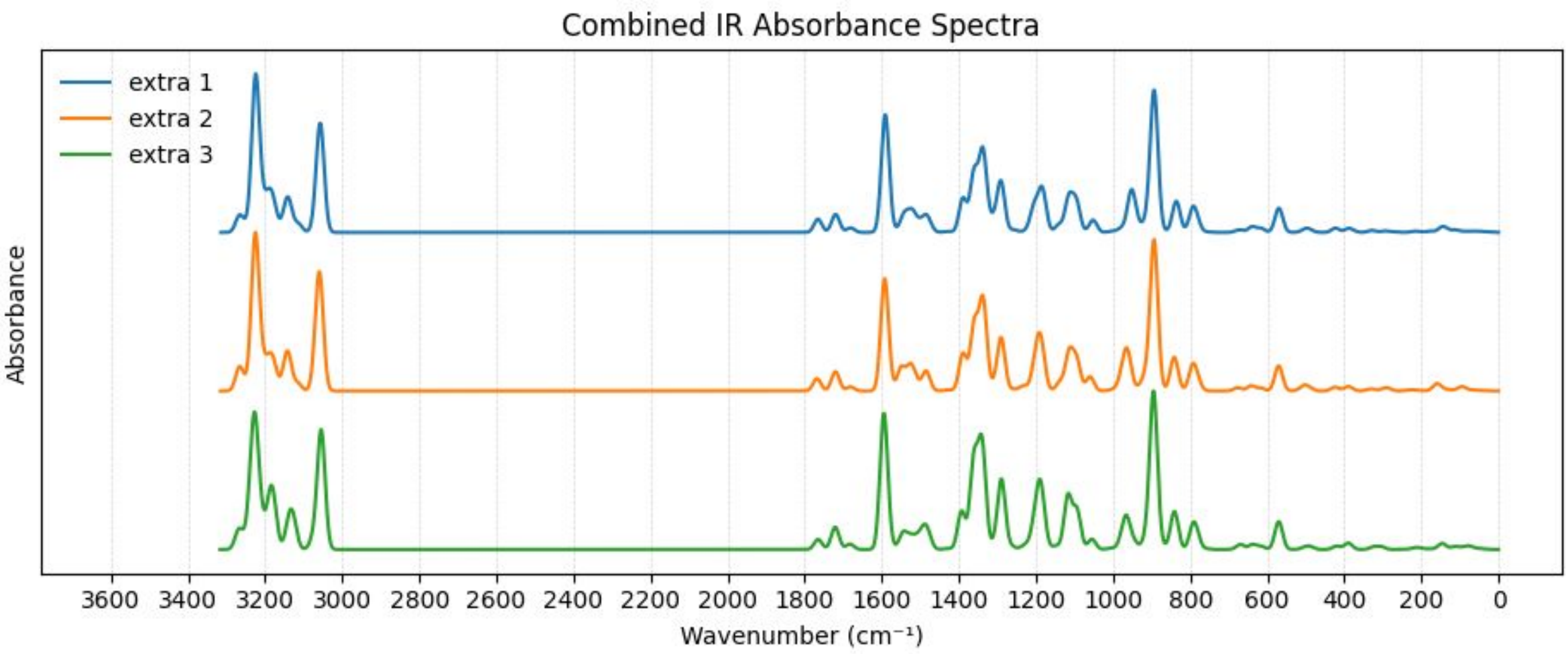

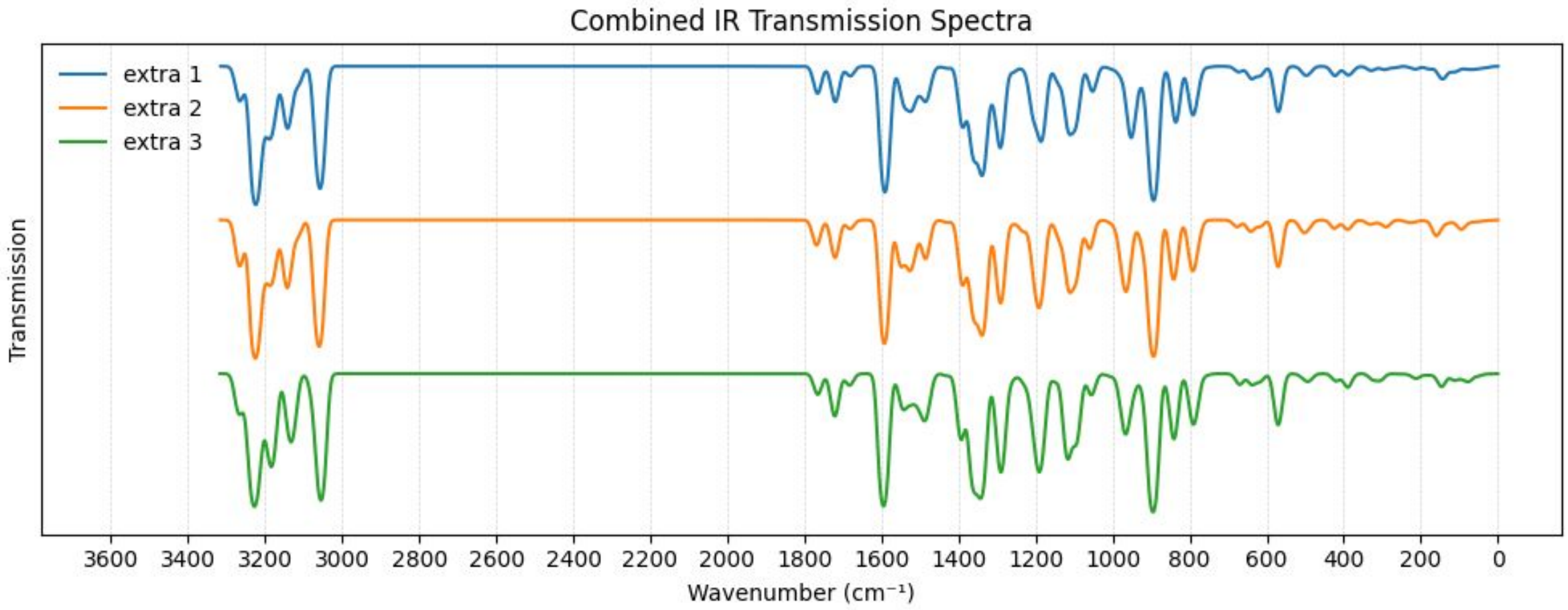

Table S7 - All energy components (E_h) were calculated for Complex 1 (Eugenol + Coronene) using the DFT/ωB97X-D3/6-31G(d), DFT/ωB97X-D3/def2-TZVPD, DFT/B3LYP-D3(BJ)/6-31G(d), and DFT/B3LYP-D3(BJ)/def2-TZVPD methods, including BSSE and BB-CP corrections.

System/Method	wB97X-D3 6-31G(d)	wB97X-D3 def2-TZVPD	B3LYP D3BJ 6-31G(d)	B3LYP D3BJ def2-TZVPD
$E_{Isolated\ Eugenol}$	-538.5334 E_h	-538.7479 E_h	-538.3953 E_h	-538.6272 E_h
$E_{Deformed\ Eugenol}$	-538.5322 E_h	-538.7469 E_h	-538.3940 E_h	-538.6261 E_h
$E_{Ghost\ Eugenol}$	-538.5359 E_h	-538.7474 E_h	-538.3984 E_h	-538.6267 E_h
$E_{Isolated\ Coronene}$	-921.6277 E_h	-921.9317 E_h	-921.4059 E_h	-921.7396 E_h
$E_{Deformed\ Coronene}$	-921.6273 E_h	-921.9315 E_h4	-921.4056 E_h	-921.7394 E_h
$E_{Ghost\ Coronene}$	-921.6305 E_h	-921.9320 E_h	-921.4095 E_h	-921.7400 E_h
$E_{Total\text{-}Complex\ 1}$	-1,460.1848 E_h	-1,460.7005 E_h	-1459,8285 E_h	-1460,3892 E_h
$E_{Ads\text{-}Complex\ 1}$	-0.0236 E_h	-0.0207 E_h	-0.0272 E_h	-0.0223 E_h
$\delta E_{BSSE\text{-}Complex\ 1}$	-0.0068 E_h	-0.0010 E_h	-0.0083 E_h	-0.0011 E_h
$E_{Ads\text{-}BB\text{-}CP\text{--}Complex\ 1}$	**-0.0167 E_h**	-0.0197 E_h	-0.018898398	-0.0211 E_h

Table S8 - All energy components (E_h) were calculated for Complex 2 (Methyleugenol + Coronene) using the DFT/ωB97X-D3/6-31G(d), DFT/ωB97X-D3/def2-TZVPD, DFT/B3LYP-D3(BJ)/6-31G(d), and DFT/B3LYP-D3(BJ)/def2-TZVPD methods, including BSSE and BB-CP corrections.

System/Method	wB97X-D3 6-31G(d)	wB97X-D3 def2-TZVPD	B3LYP D3BJ 6-31G(d)	B3LYP D3BJ def2-TZVPD
$E_{Isolated\ Methyleugenol}$	-577.8281 E_h	-578.0502 E_h	-577.6770 E_h	-577.9168 E_h
$E_{Deformed\ Methyleugenol}$	-577.8275 E_h	-578.0495 E_h	-577.6765 E_h	-577.9163 E_h
$E_{Ghost\ Methyleugenol}$	-577.8317 E_h	-578.0501 E_h	-577.6814 E_h	-577.9169 E_h
$E_{Isolated\ Coronene}$	-921.6277 E_h	-921.9317 E_h	-921.4059 E_h	-921.7396 E_h
$E_{Deformed\ Coronene}$	-921.6275 E_h	-921.9316 E_h	-921.4056 E_h	-921.7394 E_h
$E_{Ghost\ Coronene}$	-921.6310 E_h	-921.9322 E_h	-921.4101 E_h	-921.7401 E_h
$E_{Total\text{-}Complex\ 2}$	-1,499.4805 E_h	-1,500.0031 E_h	-1,499.1114 E_h	-1,499.6796 E_h
$E_{Ads\text{-}Complex\ 2}$	-0.0246 E_h	-0,0211 E_h	-0.0284 E_h	-0,0230 E_h
$\delta E_{BSSE\text{-}Complex\ 2}$	-0.0076 E_h	-0,0011 E_h	-0,0093 E_h	-0.0012 E_h
$E_{Ads\text{-}BB\text{-}CP\text{-}Complex\ 2}$	**-0.0169 E_h**	-0.0200 E_h	-0.0190 E_h	-0.0217 E_h

Table S9 - All energy components (E_h) were calculated for Complex 3 (Eugenol + Non-Hydrogenated Graphene) and Complex 4 (Methyleugenol + Non-Hydrogenated Graphene) using the DFT/ωB97X-D3/6-31G(d) method, including BSSE and BB-CP corrections.

System/Method	wB97X-D3 6-31G(d)	System/Method	wB97X-D3 6-31G(d)
$E_{Isolated\ Eugenol}$	-538.5334 E_h	$E_{Isolated\ Methyleugenol}$	-577.8281
$E_{Deformed\ Eugenol}$	-538.5319 E_h	$E_{Deformed\ Methyleugenol}$	-577.8265
$E_{Ghost\ Eugenol}$	-538.5364 E_h	$E_{Ghost\ Methyleugenol}$	-577.8306 E_h
$E_{Isolated\ Graphene}$	-913.5629 E_h	$E_{Isolated\ Graphene}$	-913.5629 E_h
$E_{Deformed\ Graphene}$	-913.5627 E_h	$E_{Deformed\ Graphene}$	-913.5626 E_h
$E_{Ghost\ Graphene}$	-913.5656 E_h	$E_{Ghost\ Graphene}$	-913.5657 E_h
$E_{Total\text{-}Complex\ 3}$	-1,452.1212 E_h	$E_{Total\text{-}Complex\ 4}$	-1,491.4153 E_h
$E_{Ads\text{-}Complex\ 3}$	-0.0248 E_h	$E_{Ads\text{-}Complex\ 4}$	-0.0242 E_h
$\delta E_{BSSE\text{-}Complex\ 3}$	-0.0073 E_h	$\delta E_{BSSE\text{-}Complex\ 4}$	-0.0072 E_h
$E_{Ads\text{-}BB\text{-}CP\text{-}Complex\ 3}$	**-0.0174 E_h**	$E_{Ads\text{-}BB\text{-}CP\text{-}Complex\ 4}$	**-0.0170 E_h**

Table S10 - The three global-minimum adsorption geometry and the corresponding energies of Complex 3 = Eugenol + Non-Hydrogenated Graphene and Complex 4 = Methyleugenol + Non-Hydrogenated Graphene using the DFT/ωB97X-D3/6-31G(d) method, including BSSE and BB-CP corrections, are presented below. The equilibrium distances between the eugenol/methyl-eugenol molecules and Non-Hydrogenated Graphene surface is in the range of 3.0–3.5 Å and 3.0–3.6 Å, respectively. Consider that 1 Hartree (E_h) = 27.2114 eV.

Systems	Geometry	Energy
Complex 3 = Eugenol + Non-Hydrogenated Graphene	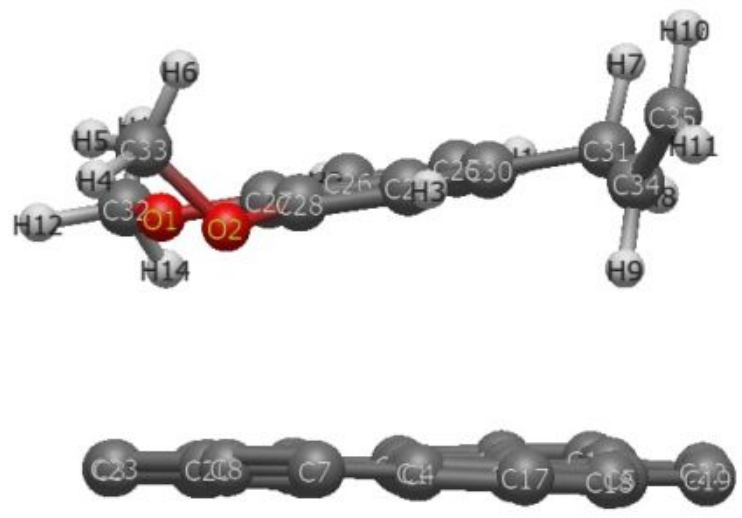	-1,452.1212 E_h<br>- 39,534.2508 eV
Complex 4 = Methy-eugenol + Non-Hydrogenated Graphene	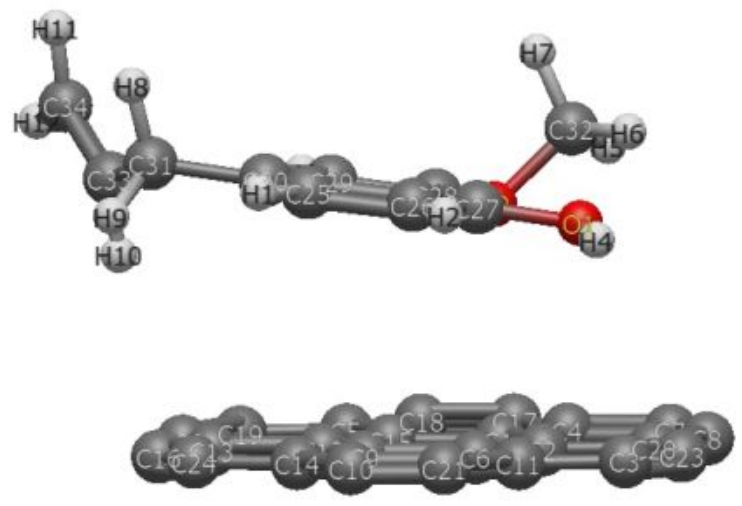	-1,491.4153 E_h<br>- 40,665.1324 eV
